# Differential exon usage of developmental genes is associated with deregulated epigenetic marks

**DOI:** 10.1038/s41598-023-38879-z

**Published:** 2023-07-28

**Authors:** Hoang Thu Trang Do, Siba Shanak, Ahmad Barghash, Volkhard Helms

**Affiliations:** 1 Center for Bioinformatics, Saarland University, Saarbrücken, Germany; 2 Department of Biology and Biotechnology, Arab American University, Jenin, Palestine; 3 Department of Computer Science, German Jordanian University, Amman, Jordan

**Keywords:** Computational biology and bioinformatics, Epigenomics, Functional genomics, RNA splicing

## Abstract

Alternative exon usage is known to affect a large portion of genes in mammalian genomes. Importantly, different splice isoforms sometimes possess distinctly different protein functions. Here, we analyzed data from the Human Epigenome Atlas for 11 different human adult tissues and for 8 cultured cells that mimic early developmental stages. We found a significant enrichment of cases where differential usage of exons in various developmental stages of human cells and tissues is associated with differential epigenetic modifications in the flanking regions of individual exons. Many of the genes that were differentially regulated at the exon level and showed deregulated histone marks at the respective exon flanks are functionally associated with development and metabolism.

## Introduction

Alternative splicing (AS) or differential exon usage (DEU) was reported to occur in 90–95% of all human multi-exon genes^1,2^ and leads to a substantial expansion of the eukaryotic proteome^3^. AS is an integral part of differentiation and developmental programs and contributes to cell lineage and tissue identity as reported by Wang et al. for nine different human tissues^4^. Based on the transcriptomes of 15 different human cell lines, the ENCODE project reported that up to 25 different transcripts can be produced from a single gene and up to 12 alternative transcripts may be expressed in a particular cell^5^.

It is well established that AS is often tightly associated with respective epigenetic chromatin modifications^6–9^. A contribution of chromatin to AS was first suggested by Adami and colleagues who found that two copies of the same adenovirus genome in the same nucleus gave rise to differentially spliced RNAs^10^. Another well-documented example where H3K36me3 influences AS of a mammalian transcript is the fibroblast growth factor receptor (FGFR2). *FGFR2* was reported by Misteli and co-workers to accumulate histone modifications H3K36me3 and H3K4me1 along the alternatively spliced region in mesenchymal cells, where exon *IIIc* is included. In contrast, H3K27me3 and H3K4me3 were found to be enriched in epithelial cells, where exon *IIIb* is used^11^. *FGFR2* is one of the rare cases where an exclusive exon switching process has been unraveled even in mechanistic terms. Precisely, in mesenchymal cells, H3K36me3 is recognized by the MRG15 protein that recruits the splicing factor PTB to the intronic splicing silencer element surrounding exon IIIB to repress its inclusion in these cells^11^. Recently, Luco and co-workers manipulated the flanks of CTNND1 exon 20 and of *FGFR2* exon *IIIb* using Crispr-Cas and showed that a single change in H3K27ac or H3K27me3 levels next to the alternatively spliced exon is necessary and sufficient to alter splicing and thereby affect EMT-related processes such as cell motility and invasiveness^12^.

Multiple studies also established a relationship between AS or DEU and differentiation or development. In 2011, Kalsotra and Cooper reviewed the roles of AS in cell division, cell fate decisions and in tissue maturation^13^. More recently, Baralle and Giudice reviewed the connection between AS and cell differentiation as well as with epigenetic landscapes, and the role of splicing processes in the brain, striated muscle and other tissues and organs^14^. More focused studies addressed, for example, how the splicing regulators Esrp1 and Esrp2 direct an epithelial splicing program that is essential for mammalian development^15^ and what role AS plays in neural development^16^. Although the pairwise connections between AS and epigenetic modifications and between AS and differentiation or development have each been characterized in detail, the intertwined connections between AS, epigenetic modifications and development have apparently received relatively little attention so far. As mentioned, Baralle and Guidice summarized some work describing such an interplay in brain and general neurological development^14^. Furthermore, an interesting study from the Heller lab related the enrichment of histone post-translational modifications (hPTMs) to AS regulation during tissue development in mice. They found, for example, that enrichment of histone modifications H3K36me3 and H3K4me1 in exon flanking regions was wired to skipped exon selection with strong evidence across all investigated embryonic tissues and developmental time points^17^.

**Figure 1:**
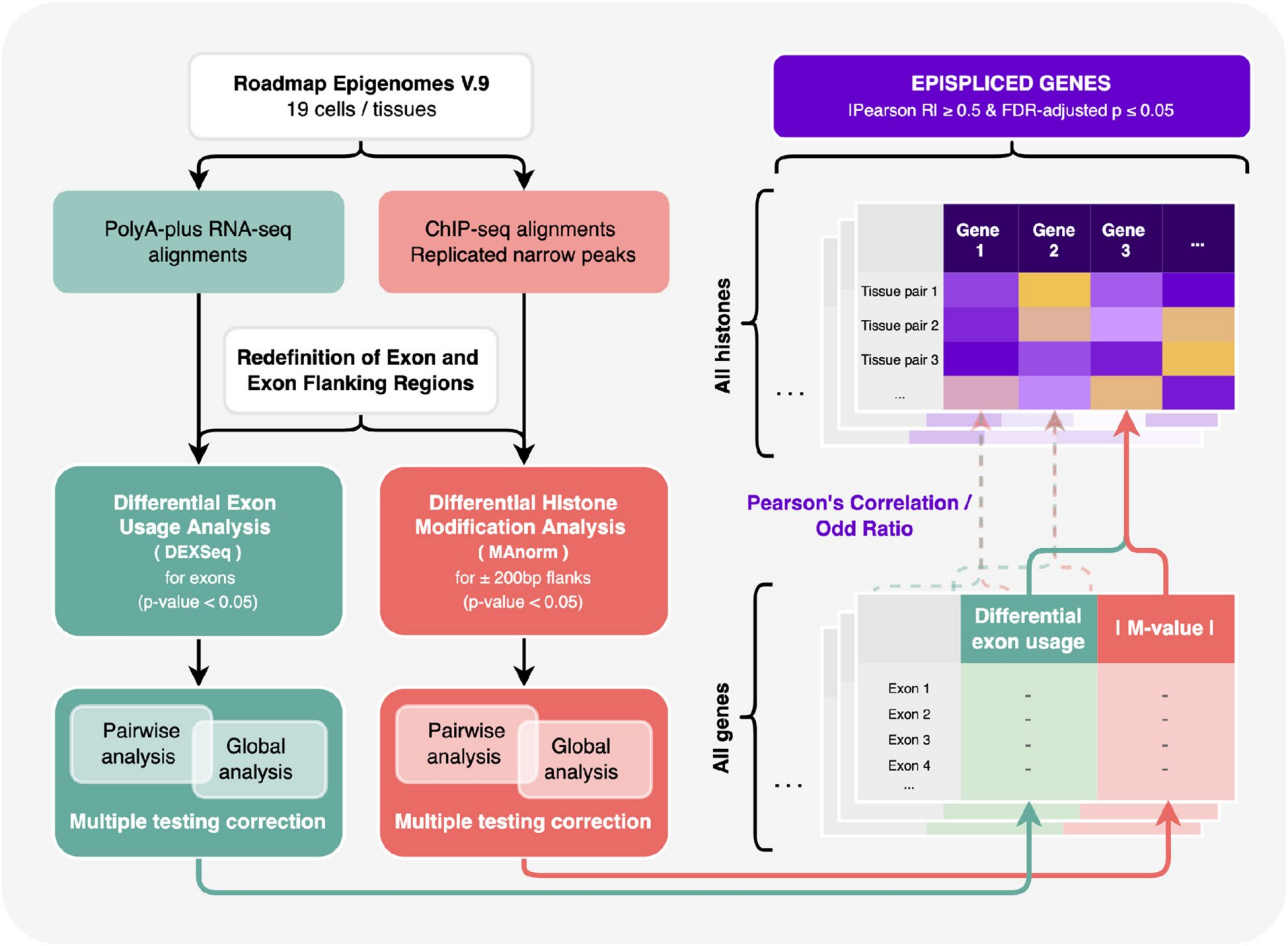
Schematic workflow to identify epispliced genes. Expression data and histone enrichment data were collected from Human Epigenomes Atlas and were subjected to differential exon usage (DEU) and differential histone modification (DHM) analysis, respectively. For each gene, we computed the Pearson correlation of the two features (DEU and DHM). Epispliced genes are required to have absolute R-value $\ge 0.5$ and FDR-adjusted pvalue $\le 0.05$. Functional enrichment analysis based on Gene Ontology terms was performed against the background of all genes having either differentially used exons or differentially deregulated histone marks at the exon boundaries.

How can one understand the postulated relationship between AS and epigenetic modifications in mechanistic terms? The most important region for epigenetically regulated AS was shown to be the exon-intron boundary. For example, Guan et al. reported strong association between epigenetic signals and cassette exon inclusion levels in both exon and flanking regions^18^. Along the same lines, flanking areas annotated with exon skipping and alternative splice site selection events were found to be statistically enriched with DNA methylation, nucleosome occupancy and histone modifications^19^. The considered exon flank should be of certain dimension, enabling a mechanistic crosstalk between a DNA position where chromatin reader proteins may recognize specific histone marks, and the downstream position on the synthesized and post-processed mRNA where splicing factors may bind. In a recent study based on ENCODE human data, Gerstein and co-workers showed that a combination of particular histone marks can be used to reliably predict using a trained machine learning classifier whether exons are included or not. Precisely, they used spatio-temporal epigenetic features extracted from exon flanks to model splicing regulation, and characterized H3K36me3, H3K27me3, H3K4me3, H3K9me3 and H3K27ac as highly influential features in the splicing regulatory model^20^. It was not the main point of our study to test again the hypothesis that one or only a few specific histone modifications mark the boundaries of either exons chosen for inclusion or exclusion from an mRNA. Instead, our manuscript focuses on which type of genes show differential splicing associated with deregulated epigenetic marks and in which context, particularly in cell fate transitions.

Based on data from the Human Epigenome Atlas^21^ for adult human tissues and cultured stem cells, we aimed at correlating differential exon usage to epigenetic modifications of different histone marks at the exon boundaries. The detailed workflow for the analysis performed in this study is illustrated in Fig. 1. Indeed, we found an overall enrichment of cases where differentially used exons overlap with differential histone marks. The involved genes were enriched in functional annotations related to the regulation of signaling and to developmental processes. When inspecting the overlap of such genes between different tissues and cell lines, we noticed a stronger overlap between cell lines corresponding to early developmental stages, whereas differentiated tissues had smaller overlaps. Besides a pooled analysis, we additionally present a detailed analysis of the two genes *FGFR2* and *LMNB1* (Lamin-B1).

## Results and discussion

### H3K36me3 mark is most relevant to AS events

The first task was to prepare a suitable data set where differentially used exons (DEUs) can be clearly associated with individual genes. Hence, out of 19,240 clusters of protein coding genes generated for the DEXSeq analysis (see “Materials and methods” section), we excluded 275 clusters of 679 genes partially overlapping with each other, 17 genes spanning more than one genomic region and 1050 genes containing only a single exon. After processing this data with DEXSeq, we filtered the detected DEUs for significance, whereby only those DEUs having a $p_{FDR} \le 0.0001$ in any of the pairwise comparisons are retained. The remaining superset of gene clusters with at least one annotated significant DEU consists of 13,837 genes. Next, we decided to focus on DEUs that were only detected in a limited number of pairwise comparisons. As revealed by the cumulative distribution in Supplementary Fig. S1A, approximately 95% of the entire DEUs library were detected in at most 25 out of 171 pairwise comparisons and are thus identified as “non-ubiquitous DEUs”. These DEUs belong to 9321 genes, which are used for the global DEXSeq analysis for multiple testing correction. In total, 10,3781 DEUs from 8887 genes were identified by both DEXSeq pairwise and global analysis. Those genes make up the final dataset of interest that will be analyzed in detail in the remainder of this study. The steps to identify these “non-ubiquitous DEUs” are summarized in Supplementary Fig. S1B.

**Table 1:** The number of detected DEU and DHM events in terms of overlap and non-overlap. DEU-DHM co-occurrence is measured by odds ratio (*OR*) with Fisher exact test (*FET*) significance and 95% confidence interval. OR was calculated as shown in Eq. (1). An *OR* greater than 1 implies a higher odd for DEU occurrence in the presence of DHM and vice versa, while *OR* of 1 indicates no association between the differential events. *FET* was used for statistical testing to determine whether the nonrandom overlap is significant ($$^{***}$$ indicates FDR-adjusted p-value $$< 0.001)$$. The 95% confidence intervals give the estimate of the precision of the *OR*s.

		Not DEU	DEU	Baseline OR	95% CI
All histones	Not DHM	8198	5149	3.68$$^{***}$$	[3.54, 3.82]
	DHM	34,585	79,888		
H3K27ac	Not DHM	30,856	55,840	1.35$$^{***}$$	[1.32, 1.39]
	DHM	11,927	29,197		
H3K27me3	Not DHM	27,103	66522	0.48$$^{***}$$	[0.47, 0.49]
	DHM	15,680	18,515		
H3K36me3	Not DHM	20,657	14,928	4.38$$^{***}$$	[4.27, 4.50]
	DHM	22,126	70,109		
H4K3me3	Not DHM	34,068	65,623	1.16$$^{***}$$	[1.12, 1.19]
	DHM	8715	19,414		
H3K9me3	Not DHM	34,079	71,022	0.77$$^{***}$$	[0.75, 0.80]
	DHM	8704	14,015		

As just mentioned, all considered genes contain at least one non-ubiquitous DEU in the epigenomes that we investigated. When all differentially modified histones are pooled, the total number of coinciding DHMs and DEUs clearly outnumbered the other three categories (Table 1). This is reflected by the total odds ratio of 3.68 (computed as $(8198 \times 79{,}888) \div (5149 \times 34{,}585)$ following Eq. (1)). However, not all considered histone marks shared high overlap with the detected AS events. In fact, only the mark H3K36me3 ($OR=4.38$) gave a pooled *OR* above 1, all the other four marks had *OR*s under or around 1 suggesting that DEUs occurred rather independently from the presence of these DHMs. This matches previous reports that H3K36me3 is most prominently associated with AS^22^.

**Figure 2:**
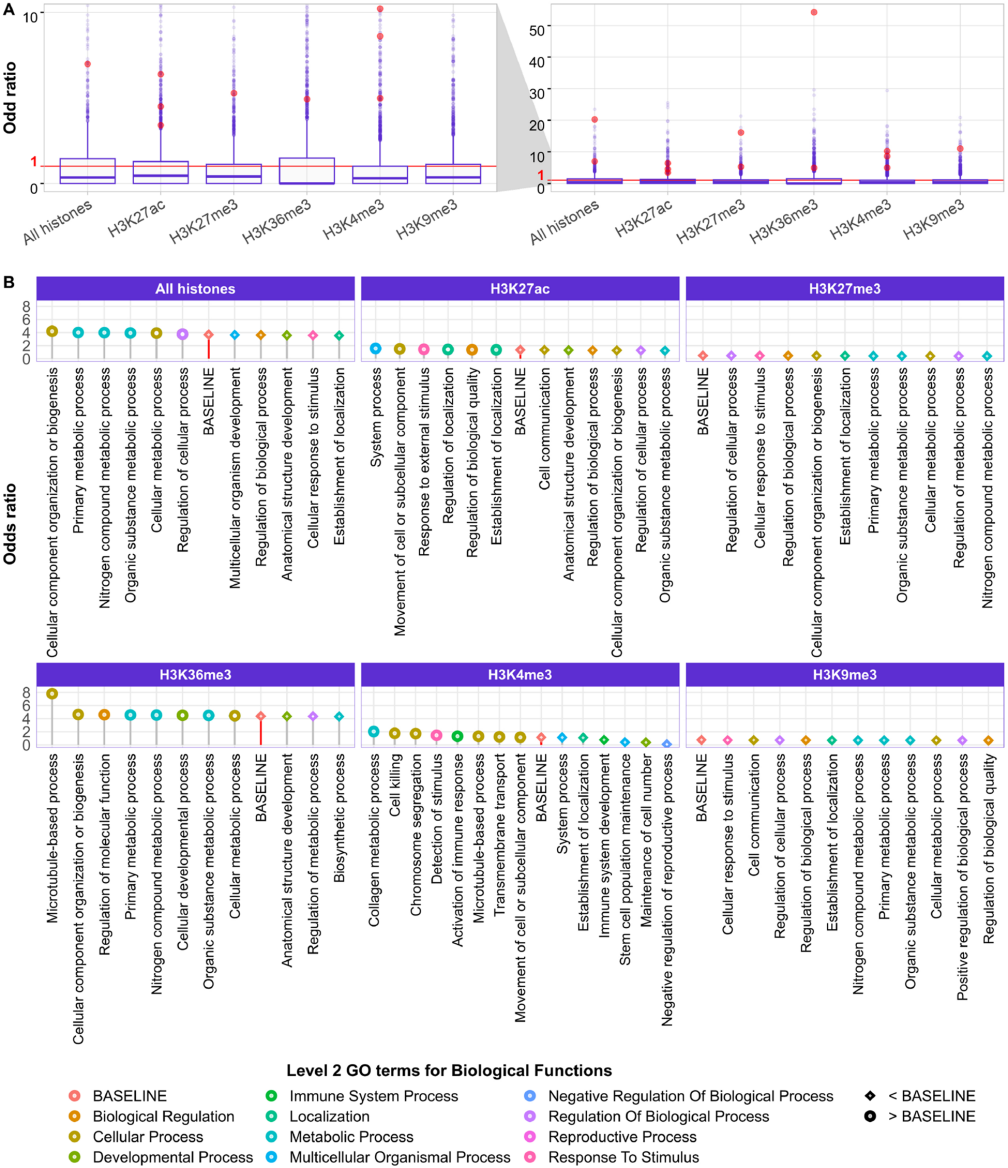
Genewise and pooled analysis of differential exon usage (DEU) and differential histone modification (DHM) co-occurrences using odds ratio (*OR*) and Fisher exact test (FET) significance. (**A**) Distribution of the *OR* for 8887 genes with at least 1 DEU and 1 DHM event, whereby genes with significant nonrandom DEU-DHM overlap ($OR \ge 1$ and $p_{FET} \le 0.05$) are highlighted in red. In (**B**), *OR* was calculated on all genes belonging to the same term at the third level of Gene Ontology (GO) terms hierarchy. These level 3 terms are colored by the GO level 2 term that they belong to. For every histone pattern, the baseline *OR* was computed based on all genes with at least 1 DHM of such histone type and 1 DEU event. The enriched level 3 terms with *OR* higher or lower than the baseline’s *OR* are denoted by round and diamond shapes, respectively.

In total, 6116 out of 8887 genes had $OR \ge 1$ (Fig. 2A). Nonetheless, after applying the necessary multiple-testing correction only 11 genes among them had a $p_{FET}$ significance below 0.05 (Supplementary Fig. S2 in Supplementary Materials). Interestingly, most of them are known to have prominent roles in cell signaling and extracellular matrix organization. Out of these 11 genes, the DEUs of two genes were associated with DHMs of all five histone marks, three genes were associated with H3K27ac, three other were associated with H3K4me3, two genes with H3K27me3, two other with H3K36me3 and one with H3K9me3. To check for a potential bias of the gene-length, Fig. S2 in Supplementary Materials plots gene-wise odds ratios as a function of exon number. Obviously, there exists a certain tendency that larger odd ratios are predominantly found for genes having fewer exons. However, the 11 genes remaining after the FET significance have quite variable numbers of exons.

We next performed the same types of analysis also for separate subgroups of genes annotated with a specific biological process term out of all level 2 or level 3 categories of the Gene Ontology. Whereas none of the subgroups annotated with level 2 terms had $OR > 1$, this was the case for several level 3 terms. Figure 2B shows odds ratios of these collective level 3 GO terms in decreasing *OR* order for each histone context. In each panel, the entries labeled as BASELINE are the same values listed in Table 1 for the superset of all considered genes. Figure 2B illustrates that most terms with higher *OR* than the baselines refer to cellular processes, localization and communication, especially for the marks H3K27ac, H3K36me3 and H3K4me3. In the scenarios where all histone marks were considered altogether or for H3K36me3 mark, the most enriched terms are associated with growth and development.

### Histone patterns and splicing decisions are tightly connected in *epispliced* genes

**Figure 3:**
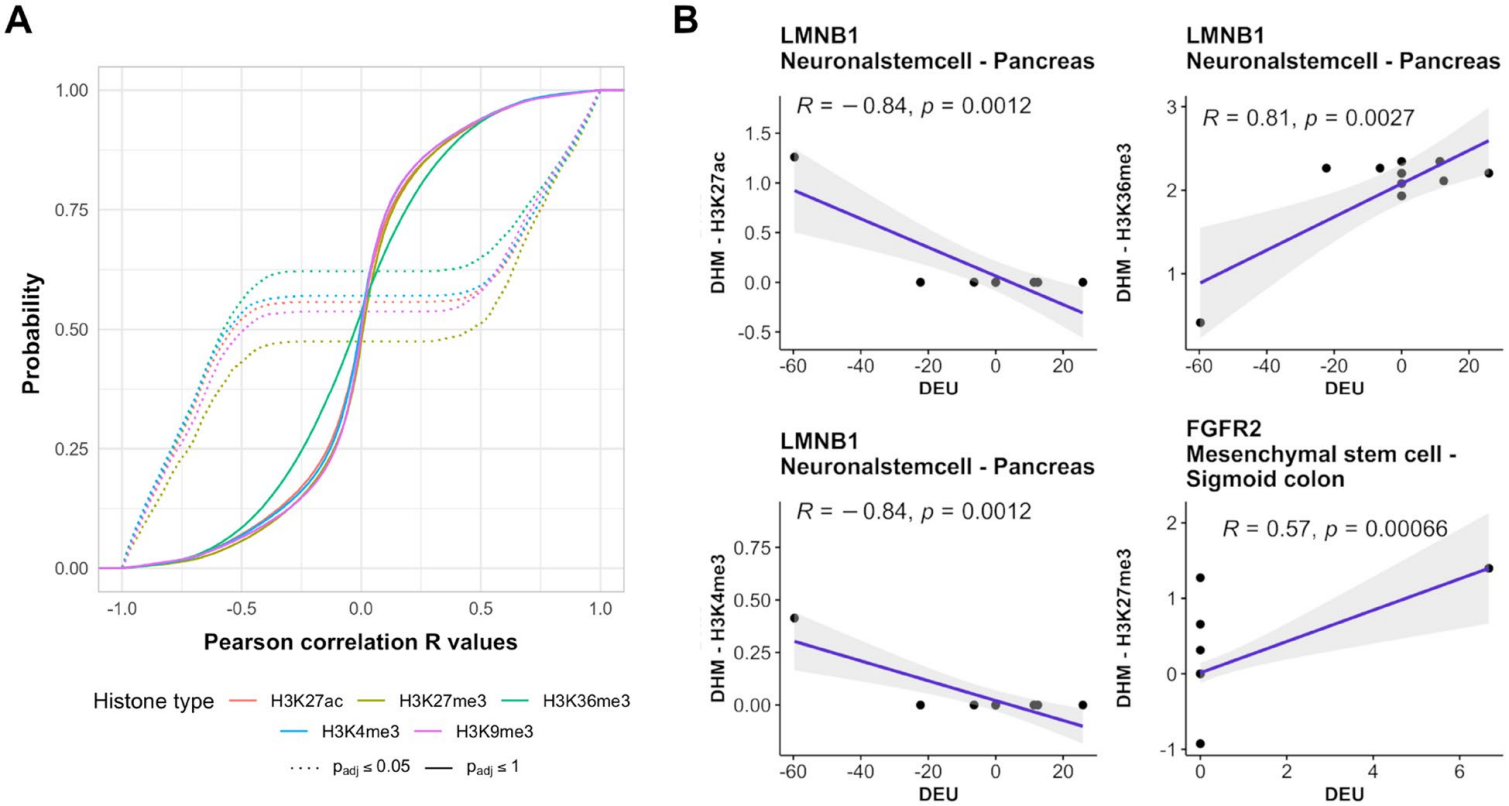
Linear association between differential exon usage (DEU) and differential histone modification (DHM). (**A**) Cumulative distribution of Pearson correlation between DEUs and DHMs for all genes. The dashed lines illustrate the cumulative distribution of all genes with the top 5% highest correlation level (FDR-adjusted p-value = 0.05). (**B**) Pearson correlation between differential exon usage (detected by DEXSeq) and deregulation of histone marks ($M-value$s detected by MAnorm) for the two genes *LMNB1* and *FGFR2*, respectively. For *LMNB1*, exon usage and histone modification were compared between neuronal stem cell and pancreas, and between mesenchymal stem cell and sigmoid colon for *FGFR2*, respectively.

As previously shown, differential placement of chromatin marks has a substantial impact on post-transcriptional processes including alternative splicing^17,18,23,24^. With the aim of delineating their role in human development, we now identified those genes where differential exon usage is linearly correlated to the degree of histone mark deregulation at the exon boundaries. These regions, alternatively referred to as “flank” or “flanking regions”, were defined as a span 200-bp up- or downstream from the exon start or end points as suggested in related studies (Fig. 7B)^17,23,25^. Based on the analysis of odd ratios presented above, we conclude that there exists in fact a significant association between DEU and DHM at least for a fraction of genes. Only the top 5% of the investigated genes had a DEU-DHM correlation higher than the absolute Pearson correlation coefficient $|R| =0.5$, see (Fig. 3A). Hence, we used this value as suitable threshold to identify “epispliced” genes.

**Figure 4:**
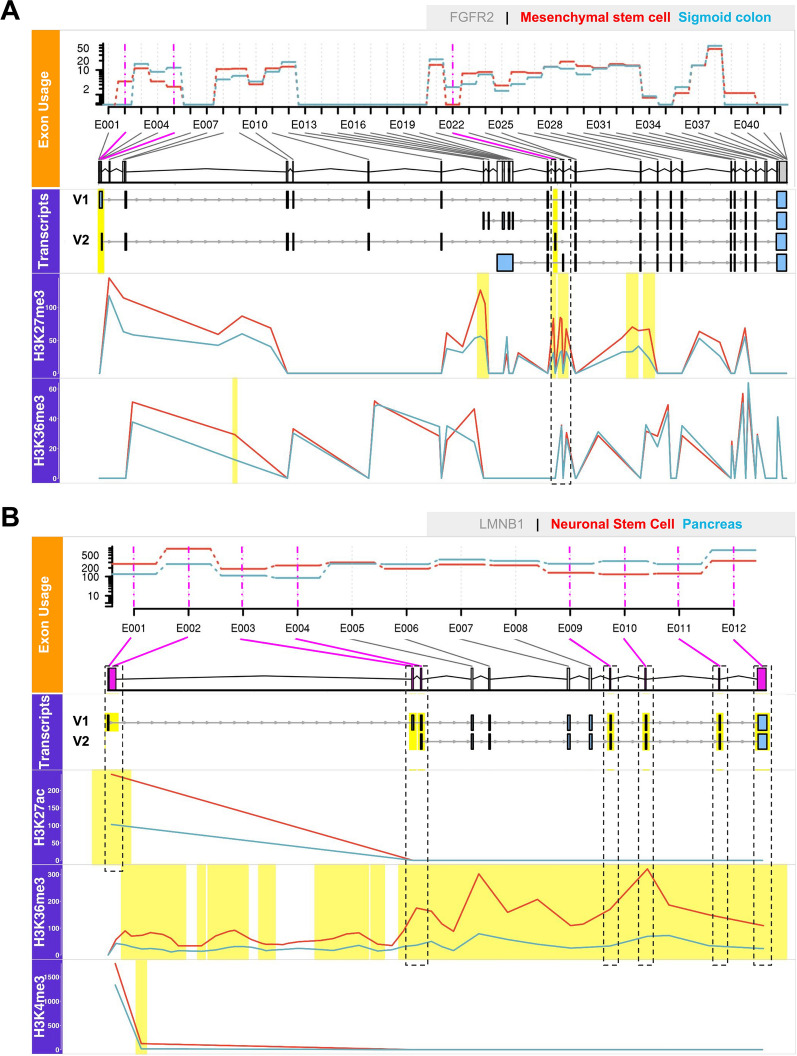
Two case studies (*FGFR2* and *LMNB1* genes) of deregulated epigenetic modifications associated with alternative splicing. The upper, orange-labeled panels that illustrate exon usage were produced by the DEXSeq package. In this case, they highlight the differential exon usage (DEU) of the *FGFR2* gene between mesenchymal stem cell and sigmoid colon (**A**), and of the *LMNB1* gene between neuronal stem cells and pancreas (**B**). Significantly differentially used exons (FDR-adjusted p-value $p_{FDR} \le 0.05$) are marked in pink. The panels shown below that are colored in violet illustrate the association of DEUs and epigenomic modifications for the same two genes and tissues. Regions highlighted in yellow represent exons with DEUs identified from DEXSeq ($p_{FDR} \le 0.05$) and significant differentially abundant peaks of histone modifications detected by MAnorm ($p_{FDR} \le 0.05$ and |M-value$| \ge 1$). The boxes in the “Transcripts” panel show transcript variants found in the investigated cell types as retrieved from NCBI Refseq. The figure was generated using the Gviz package.

As examples, Fig. 3B shows the Pearson correlations for the gene *FGFR2* in mesenchymal stem cells against sigmoid colon tissue and for *LMNB1* in neuronal stem cells against pancreas tissue. For *FGFR2*, AS events and splicing mechanisms have been frequently discussed^7,8,11,13^. We found that DEU of *FGFR2* was positively correlated to H3K27me3 DHMs with a coefficient of 0.57. One may question whether the DEU-DHM correlation plot for *FGFR2* (Fig. 3B) represents a meaningful linear relationship. We note, however, that generally DEU only affects at most 3 exons of a gene (43.30% of all DEU cases detected from 171 pairwise comparisons). As a result, correlation plots such as the one shown for *FGFR2* are quite common. In this plot, the non-zero correlation is basically due to one point with high DHM and high DEU values. Note, however, that our DEU-DHM association analysis is based on data measured for multiple samples each and we only consider values that remained after statistical significance testing. Hence, this point does not represent an outlier that typically confuses Pearson correlation analysis, but it is a true data point. Those points having $DEU = 0$ but different DHM values are typical non-DEU exons where epigenetic deregulation may also affect other processes. As second example, we show the gene *LMNB1* which had relatively high correlations between DEUs and DHMs and this was the case for 3 out of 5 considered differential histone marks ($R = -0.84, 0.81$ and $-0.84$ for H3K27ac, H3K36me3 and H3K4me3, respectively), which only occurred for a few genes. For comparison, Podlaha et al. reported Spearman rank correlations of protein-coding genes between H3K36me3 enrichment level and splicing exon inclusion rate of at most 0.36 for six normal human cell lines^22^. From now on, we will use the term “epispliced genes” to refer to genes showing significant absolute correlations greater than 0.5 (threshold obtained from $p_{FDR} \le 0.05$, Fig. 3A). For clarity, we accompany Fig. 3B with a more detailed representation of the transcript architecture of the same two genes *FGFR2* and *LMNB1* in Fig. 4.

#### Case study 1: *FGFR2* gene

For *FGFR2*, DEUs between mesenchymal stem cells and sigmoid colon were initially detected for exons 2, 5 and 22. These exon numbers refer to a flattened exon model used for the DEXSeq analysis. However, exons 2 and 5 were subsequently excluded from the analysis since they are the first exons of several transcript variants. The only detected AS event in this comparison was the exon skipping at exon 22 in mesenchymal stem cells as shown in the orange panel for exon usage of Fig. 4A. When mapped to the NCBI reference genome, exons 21–23 correspond to exons IIIa, *IIIb* and *IIIc* discussed in previous studies^26^. In fact, those three exons are known to determine the two most prominent, mutually exclusive transcripts of *FGFR2*, namely *FGFR2b* and *FGFR2c* (as shown by V2 and V1 transcripts in the “Transcripts” panel of Fig. 4A). The inclusion of exon *IIIb* (exon 22 in our annotation) and exclusion of *IIIc* (exon 23) give rise to the epithelial-specific *FGFR2b* variant, whereas the opposite case results in the mesenchymal-specific *FGFR2c* variant^15^. Using DEXSeq, we found strong evidence for the dominance of *FGFR2c* in mesenchymal stem cells and of *FGFR2b* in sigmoid colon tissue. Meanwhile, MAnorm detected a significantly higher H3K27me3 signal in mesenchymal stem cells (red) at the flank regions of exons *IIIb* and *IIIc* that in fact coincides with the recent experimental findings by Luco and coworkers^12^. These authors also reported anti-correlation between the inclusion level of exon *IIIc* and the localized enrichment level of H3K27me3 during epithelial-mesenchymal transition that is evident in our comparison between mesenchymal stem cells and sigmoid colon (Fig. 4A—dashed black box). Additionally, the enrichment of the H3K27me3 mark at the *FGFR2* promoter has also been linked to the down-regulation of exon *IIIb*^27^. In their previous study, Luco et al. reported an enrichment of H3K36me3 over the length of the *FGFR2* gene that is linked to exon *IIIb* skipping in mesenchymal stem cells^7,11^. They speculated that the histone mark represses exon inclusion by recruiting two RNA-binding proteins MRG15 and PTB to the splice sites. Here, even though such enrichment can be observed in the last panel, we did not find a significant correlation between differential modification of H3K36me3 and *FGFR2* alternative exon usage. Nonetheless, it has recently been confirmed experimentally that the localized H3K36me3 mark rarely showed correlation to the changes in exon *IIIc* inclusion level^12^. This good match with experimental findings for individual well-studied genes emphasizes the importance of genome-wide examination of histone modification in AS contexts as is done here.

#### Case study 2: *LMNB1* gene

For *LMNB1* (Fig. 4B), two transcript variants including NM_005573 and NM_001198557 are presented as V1 and V2 in the “Transcripts” panel. While the first variant produces an isoform that includes all presented exons, the latter yields a shorter isoform consisting of only exons 4–12 due to a different 5$^\prime$ UTR^28^. Here, the exons 1–4 and 9–12 are clear examples of strong differential exon usage between neuronal stem cells and pancreas. All these exons also showed significantly modified histone patterns at their flank regions as highlighted by the black boxes. Since we decided to exclude all first exons of any annotated transcript to cast aside any transcription-related histone signals, the left-most box encloses only exon 2. As mentioned above, the H3K27ac, H3K27me3 and H3K36me3 marks are significantly correlated to DEU for *LMNB1*. Figure 4B shows these marks in the three lowest rows. The two marks H3K27ac and H3K4me3 are more pronounced around the boundaries of exon 2 that have an elevated exon usage in neuronal stem cells. Furthermore, the H3K36me3 level in neuronal stem cells (red) is higher than in pancreas (blue) at exons 6–12, which intriguingly overlaps with the lower exon usage in neuronal stem cells. Considering that the elevated usage of these exons might signify a higher abundance of the shorter variant of *LMNB1* (NM_001198557) in pancreas, the histone mark H3K36me3 could serve a substantial role in the selection of alternative isoforms in these cell types.

### Histone modification influences alternative splicing in developmental genes

The main biological aim of this paper was to investigate a possible relationship between episplicing and development. Thus, we were less interested in detecting genes that are alternatively spliced in a similar manner in many pairwise epigenome comparisons. Rather, we focused on those genes showing differential exon usage coupled to epigenetic rewiring in relatively few tissue comparisons. Such a subset of genes is captured by filtering for the least ubiquitously occurring DEUs (see “Materials and methods” section). Table 2 shows the subsets of *epispliced* genes which have DEU events that were detected in a limited number of tissue comparisons (1–25 out of 171). If we find such an event in the comparison of two tissues A and B, this event is counted both for A and B. Sometimes, a gene may show correlated DEU and histone mark levels for multiple histone marks. The last column lists the total number of non-ubiquitous *epispliced* genes where these overlaps are omitted. As mentioned before, these genes contain the non-ubiquitous DEUs that appeared in a limited number of pairwise tissue comparisons. The three stem cells including neuronal stem cells, H1 stem cells and mesenchymal stem cells featured the largest number of non-ubiquitous *epispliced* genes, whereas aorta and esophagus are among the ones having the fewest of such genes (together with psoas muscle and sigmoid colon).

**Table 2:** Number of “epispliced” genes with non-ubiquitous DEU events across all cell types in different epigenomics contexts. To account for non-ubiquitous exons, the genes with alternative splicing events occurring in a limited number of (1–25) tissue comparisons were selected from the differential exon usage analysis. “Epispliced” genes are genes where exon inclusion is correlated to differential modification of either H3K27ac, H3K27me3, H3K36me3, H3K4me3 or H3K9me3. The two rightmost columns list the count of “epispliced” genes with or without inclusion of repeating cases. (–) denotes cases where ChIP-seq histone peaks data was not available.

Tissue	H3K27ac	H3K27me3	H3K36me3	H3K4me3	H3K9me3	Total number of “epispliced” genes (with overlaps)	Total number of “epispliced” genes (without overlaps)
Adipose tissue	425	–	–	–	–	425	425
Aorta	414	166	383	324	136	1423	1125
CD4-positive alpha beta T cell	717	310	658	554	377	2616	1875
CD8 positive alpha beta T cell	750	241	648	548	302	2489	1843
Ectodermal cell	791	286	–	575	277	1929	1463
Endodermal cell	799	315	683	630	449	2876	2073
Esophagus	470	169	420	354	198	1611	1241
H1 cell	977	538	832	677	449	3473	2457
Mesenchymal stem cell	931	333	744	679	369	3056	2264
Mesendoderm	943	271	838	667	–	2719	2033
Mesodermal cell	727	–	651	490	312	2180	1688
Neuronal stem cell	963	435	816	822	621	3668	2611
Pancreas	593	221	629	405	295	2143	1646
Psoas muscle	544	202	485	403	165	1799	1363
Sigmoid colon	517	193	480	321	164	1675	1313
Small intestine	625	223	552	338	225	1963	1483
Spleen	491	201	530	367	302	1891	1484
Stomach	532	254	530	364	275	1955	1513
Trophoblast	942	305	790	–	369	2406	1963

**Figure 5:**
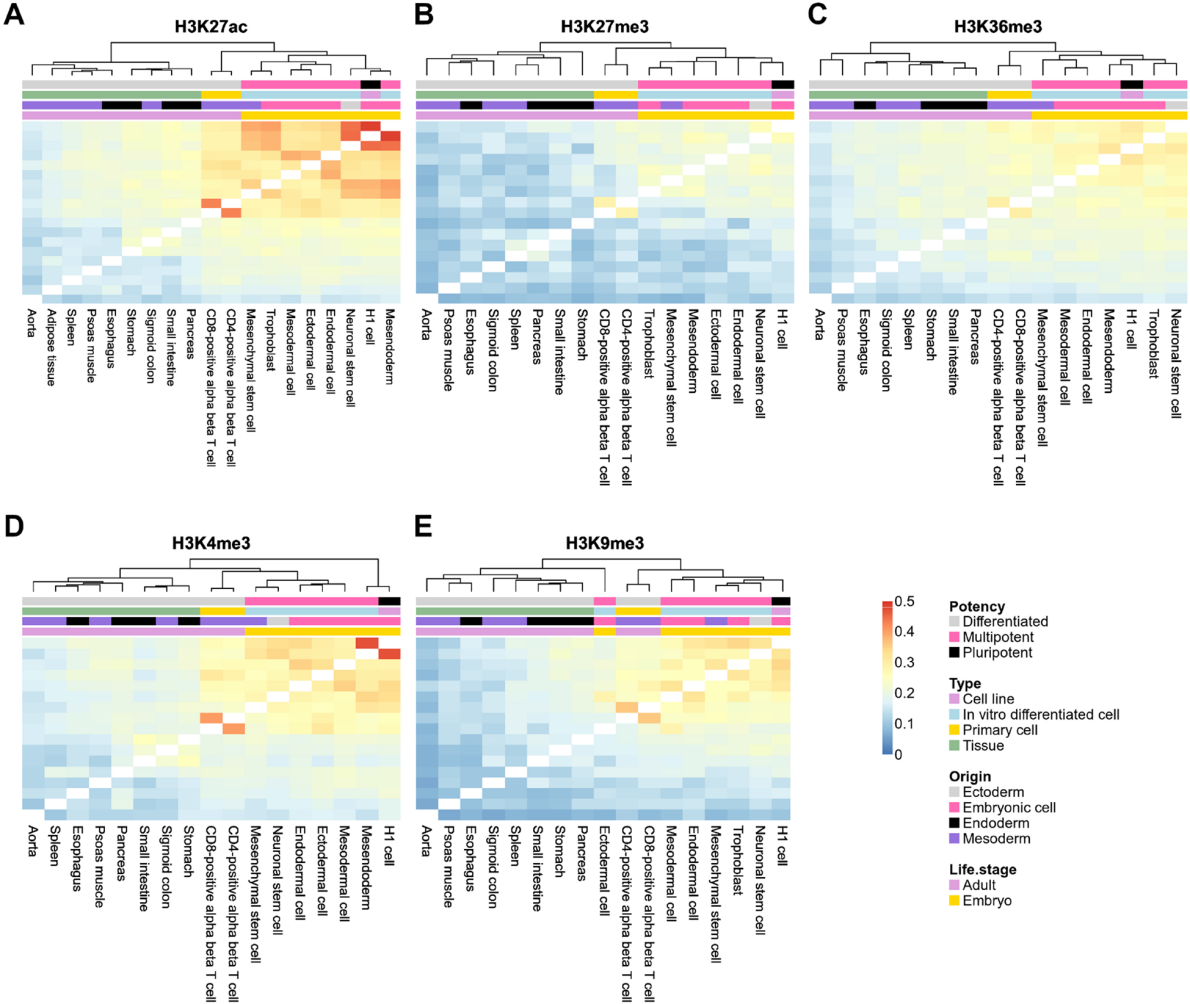
Heatmaps representing hierarchical clustering based on the similarity in non-ubiquitous “*epispliced*” genes in different epigenetic contexts. In total, 19 cell types were considered for H3K27ac, H3K27me3, H3K36me, H3K4me3 and H3K9me3 (**A**–**E**). The pairwise similarity between cell types was measured by the Jaccard index, which is the ratio between the number of mutual *epispliced* genes and the total number of *epispliced* genes in the union sets of two cell types (Eq. 2). All heatmaps use the same color scale ranging from 0 to the highest Jaccard index across all tissue pairs and for different histone marks. Investigated epigenomes were annotated on the top by their differentiation potency, type of sample, germ layer origin and the life stage when their samples were taken.

After identifying *epispliced* genes for individual epigenomes, we analyzed which cell types shared the most or fewest non-ubiquitous *epispliced* genes. For this, we computed pairwise similarities between epigenomes by taking their Jaccard index (Eq. 2) based on the non-ubiquitous *epispliced* genes listed in Table 2. As an example, the largest overlap of 628 shared non-ubiquitous *epispliced* genes exists between H1 cells and mesendoderm, while their union of non-ubiquitous *epispliced* genes is 1292 genes. This then gives a Jaccard similarity of 0.486 (Fig. 5A). The similarity values are generally not remarkably high, reflecting clear differences in isoform expression between any two cell types. On the other hand, there are also clear similarities between certain epigenome pairs. Thus, for each mark, we applied hierarchical clustering to group cell types with higher similarities into clusters. As the same time, the epigenomes were described in four ways: by potency, sample type, origin and life stage. To quantify which labeling type was associated most strongly with the clustering obtained, we computed adjusted Rand indices that quantify how well the labeling scheme matches the clustering results (Table 3).

Figure 5 shows a clustered heatmap of the similarity of non-ubiquitous *epispliced* genes between pairs of epigenomes. For H3K27me3 (panel B) and H3K9me3 (E), only relatively small similarities were found between all cell types. For the H3K27ac mark (panel A), the largest similarities were found between neuronal stem cells, H1 cells and mesendoderm as well as between CD4 and CD8 immune cells. Differentiated tissues showed again rather low similarities among each other and with multipotent and pluripotent cells. For all histone marks, samples belonging to the same type shared most non-ubiquitous *epispliced* genes with relatively high Rand indices ranging from 0.703 to 0.911 (Table 3). This is reflected by the fact that all differentiated tissues were clustered together. Among these, the tissue pair CD4 and CD8 cells always shared the highest similarity. We also observed a cluster of six pluripotent and multipotent cells (neuronal stem cells, H1 cells, trophoblast or ectodermal cell, mesendoderm, mesodermal cell, endodermal cell) sharing fairly high similarity in all histone contexts, especially for H3K27ac (A). This matched the Rand indices that show high clustering purity according to potency and life stage (0.518 and 0.602) for this mark. For those two categories, the clusters in H3K9me3 were dissimilar to those found from other histone modifications, as demonstrated by the low Rand indices for potency, origin and life stage (Table 3).

**Table 3:** Adjusted Rand indices measuring the similarity between heatmap hierarchical clustering and tissue label schemes. Investigated cell types were separated by potency, sample type, origin and life stage and compared to the cluster labels from hierarchical clustering, separately for differential exon usage correlated with the five histone modifications labeled in the table header. The second row lists the number of cell types analyzed for each histone mark.

	H3K27ac	H3K27me3	H3K36me3	H3K4me3	H3K9me3
Number of available cell types	19	17	17	17	17
Potency	0.518	0.467	0.467	0.467	0.319
Type	0.911	0.903	0.703	0.740	0.711
Origin	0.349	0.314	0.314	0.181	0.160
Life stage	0.602	0.558	0.558	0.558	0.381

Overall, stem cells and multipotent cells shared the largest number of non-ubiquitous *epispliced* genes especially for the two histone marks H3K27ac and H3K4me3, whereas differentiated cells tended to have rather low similarities for all five histone marks. The only exceptions to this were the immune cell types CD4 and CD8 that also had high similarities for H3K27ac, H3K4me3 and H3K9me3. One may wonder if analyzing shared DEU or DHM events alone would yield a similar clustering of tissues. This is analyzed in Fig. S3 and Table S6 in Supplementary Materials. Obviously, the clustering based on either DEU or DHMs does not produce a meaningful clustering and gives only lower-valued Rand indices. In our view, this emphasizes the value of performing an integrative analysis of shared DEU and DHM events as is done in Fig. 5.

**Figure 6:**
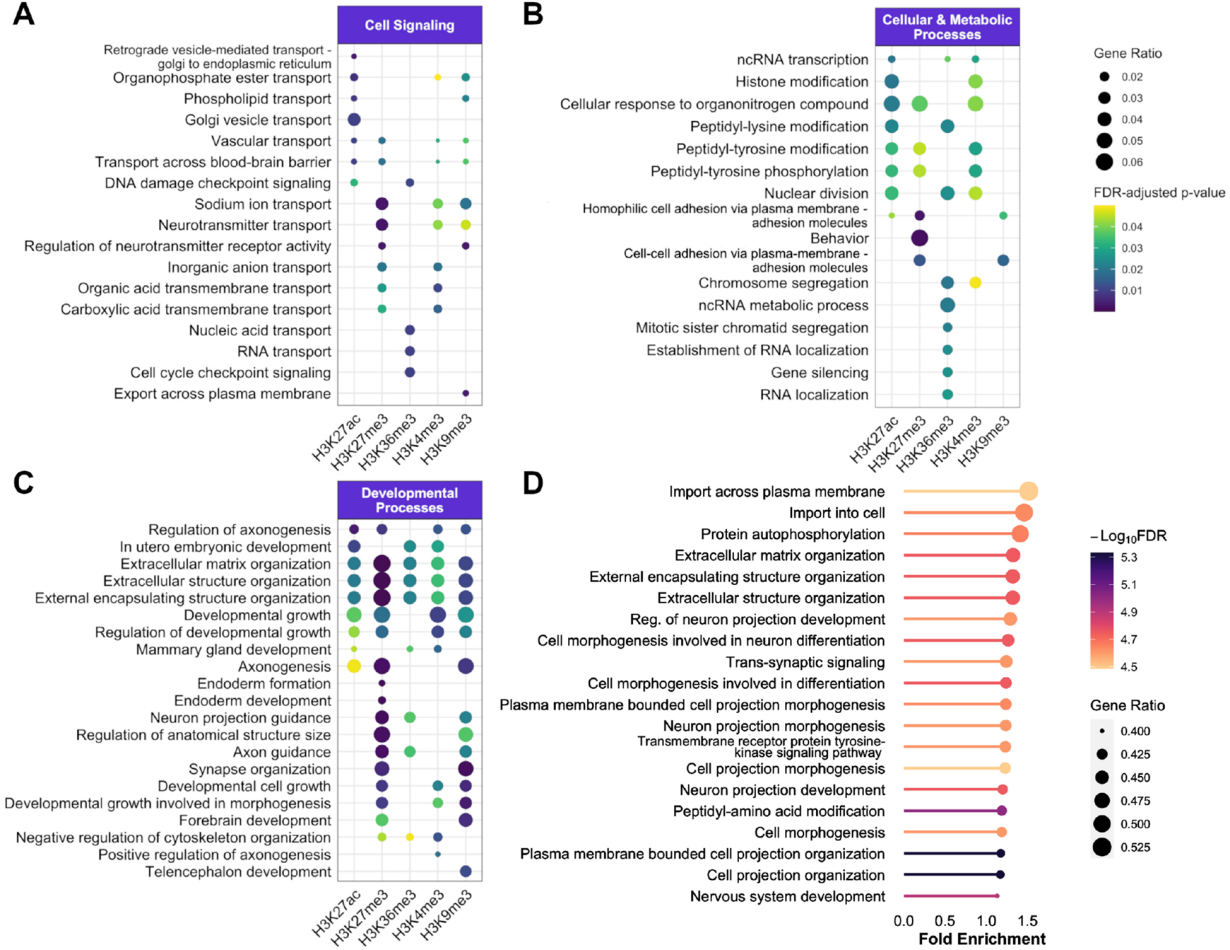
Gene ontology (GO) enrichment analysis for biological functions of non-ubiquitous *epispliced* genes for each histone type. The top enriched GO terms (FDR-adjusted p-value $\le 0.05$) annotated to *epispliced* genes that were correlated either with H3K27ac, H3K27me3, H3K36me, H3K4me3 or with H3K9me3 differential histone modifications were sorted in decreasing order of significance and of mutual functions between the histone marks. The GO terms are grouped into three main categories, namely cell signaling (**A**), cellular and metabolic processes (**B**) and developmental processes (**C**). (**D**) Shows the terms enriched for the union set of *epispliced* genes detected from all histone contexts in decreasing order of fold enrichment. In the enrichment analysis, the respective *epispliced* gene sets were compared against the background set of all genes having either differentially used exons or differentially deregulated histone marks at the exon boundaries.

Finally, we performed functional enrichment analysis of the non-ubiquitous *epispliced* genes separately for each histone mark. Figure 6 shows the results of gene-set enrichment analysis based on the Gene Ontology annotations of *epispliced* genes. The terms are arranged into three broad GO-SLIM categories, including cell signaling, developmental processes and cellular/metabolic processes. It turned out that the category of developmental processes played a dominant role with the highest number of terms shared between *epispliced* gene sets of different histone marks (Fig. 6C). The mark H3K27me3 seemed to have the largest contribution in this. Coincidentally, H3K27me3 also gave the second clearest separation according to sample types and origins of investigated tissues ($Rand\ indices = 0.903$ and 0.314, respectively). In a similar GO term enrichment analysis performed on the set of *epispliced* genes with correlation and anticorrelation separately, many of these biological annotations are found to associate with the direction of histone mark deregulation (Fig. S4 in Supplementary Materials).

The functional annotations related to the H3K27ac and H3K27me3 histone marks had the largest overlap of developmental GO terms at level 3 hierarchy (Fig. 2B). Besides, H3K27ac yielded the highest purity in clustering the tissues by potency, sample type, origin and life stage ($Rand\ indices = 0.518, 0.911, 0.349, 0.602$, respectively). On the other hand, the GO terms in other categories of H3K27ac and H3K27me3 had little in common: “Epispliced” genes with deregulated H3K27me3 marks were mainly enriched with cell signaling functions (Fig. 6A), while those with deregulated H3K27ac marks were rather involved in cellular or metabolic processes, specifically in post-translationational modification (Fig. 6B). Another histone mark contributing prominently to the developmental category was H3K9me3 with many unique GO terms related to systemic development. Indeed, these results appear to have much clearer biological consequences than our initial analysis of DHM-DEU overlaps based on *OR*s, which did not show significantly enriched biological functions for many histone marks, especially for H3K27me3 and H3K9me3 (Fig. 2B). For the two marks H3K36me3 and H3K4me3 which shared less similarity in GO terms with others, epigenetic regulation of differential exon usage was also important for several rather general metabolic and signaling processes. Interestingly, these *epispliced* genes with non-ubiquitous DEU events also had important roles specifically in post-translational modification of proteins (Fig. 6B). Upon considering the direction of DHM-DEU relationship, we also found that most of the development-related terms were enriched for genes where exon usage was anti-correlated to transcriptional silencing marks H3K27me3 and H3K9me3 or correlated to activation marks H3K27ac and H3K4me3 (Supplementary Fig. S4 in Supplementary Materials). An exception to this observation is the set of genes enriched in extracellular matrix organization which were associated with both suppressed and enhanced histone modification signals. We furthermore noticed the lack of enriched terms for transcriptional processes, despite the evident influence of histone modification on transcription^25^. This effect likely resulted from our decision to remove the first exons of any transcript variant from our analysis.

The same type of functional enrichment analysis was also carried out for the union set of *epispliced* genes detected across different histone modification contexts. The result of such an analysis revealed that cell morphogenesis and neurogenesis sub-processes have the highest fold enrichment after cell import and protein autophosphorylation (Fig. 6D). Again, the enriched terms for combined histone marks contained more significant and development-centric GO terms than those from the DEU-DHM co-occurrence analysis (Fig. 2B). One should note that other studies have already linked such histone pattern alterations to developmental processes. For instance, genes with H3K27ac-enhanced regions have been previously associated with GO functions that are characteristic for multipotent stem cells, such as anatomical structure development and nervous system development^29^. Broad H3K4me3 domains were also reported to have distinctive roles in neuronal development during stem cell and human brain tissue differentiation, which is in concordance with our findings^30^. Furthermore, the H3K4me3 and H3K27me3 promoter bivalency was established as a prominent epigenetic mechanism for lineage-specific activation or repression of developmental genes in embryonic and neural stem cell differentiation^31,32^. For H3K36me3, we described that many GO terms contributed to cellular component organization or RNA processing and regulation besides morphogenesis, which opens up the possibility that the histone mark contributes to developmental processes via transcriptional regulation. In mouse embryonic stem cells, crosstalk between H3K36me3 and the RNA modification m6A mediates the maintenance of pluripotent state and initiates differentiation via recruitment of RNA methyltransferase complexes^33^.

Finally, we add a word of caution about a possible limitation of our study where we mixed data from cell lines with data from tissues. Grouping data by “type” indeed gave rather high Rand indices in Table 3. Interestingly, this was not the case when clustering was based on shared DEUs or DHMs alone (Table S6 in Supplementary Materials), which speaks against a general bias of this mixing approach. We agree that, ideally, all data should either come from cell lines or from tissues. Unfortunately, to our knowledge such data is currently not publicly available. In future, a similar type of analysis could possibly be done based on single-cell data.

## Conclusions

Epigenetic histone marks at the exon-intron boundaries do not only play a role in defining the elements for the mRNA transcript to be expressed. Rather, as shown before, they can also contribute to regulating and controlling the relative abundance of different transcripts or protein isoforms that map to the same chromosomal region across tissues. Here, this relationship was captured by identifying genes where exon usage and histone marks at the exon flanks show concerted differential changes. We showed that there is a global enrichment of simultaneous differential exon usage and differential histone marks that is statistically significant for different subgroups of developmental genes. Taking *FGFR2* and *LMNB1* as examples, we highlighted exon-intron junctions as hot-spots for local epigenetic modifications which potentially have roles as splicing regulatory elements. Furthermore, we observed that the relationship between differentially used exons and differentially modified histone marks seems to be most prominent in early embryonic development, which suggests differential regulation across developmental stages. While this finding applied to the five studied histone marks, our assessment of *epispliced* genes also revealed further biological roles annotated to such genes for individual modification patterns. “Epispliced” genes related to H3K27me3 and H3K9me3 are mainly involved in cell signaling processes. On the other hand, the alternatively spliced genes associated to H3K27ac, H3K36me3 and H3K4me3 are potential key factors in chromatin remodeling and post-translational protein modifications, which in turn reinforce the epigenetic regulation of transcriptional and splicing activities.

## Materials and methods

### Data preparation

#### Transcriptomic and epigenetic data sets from the Human Epigenome Atlas

We examined the association between the differential usage of exons and epigenetic marks using RNA-seq and ChIP-seq data for histone modifications from the Human Epigenome Atlas (release 9)^21^. The data belongs to the Roadmap Epigenomics Project^21^ and was downloaded from the ENCODE portal^34^ at https://www.encodeproject.org/ for the histone marks H3K27ac, H3K27me3, H3K36me3, H3K4me3 and H3K9me3. Cells or tissues that either lacked biological replicates, were flagged for poor quality controls, or had unclear developmental origin were excluded from the study. For the sake of homogeneity, only embryonic and adult samples were considered. In total, we analyzed 19 epigenomes including one cell line, seven in vitro differentiated cells, two primary cells and nine tissues passing the described filters, each with minimum 2 and maximum 5 biological replicates. The samples were categorized by their potency, the life stage at their harvest time and the germ layer from which they arise. Table S1 in Supplementary Materials lists the tissues and cell lines included in the current analysis, while metadata reporting all retrieved samples in details with regard to sources, biosample types and used parameters for bio-assays can be found in Tables S2–S4.

#### Annotation of gene body and flank regions

**Figure 7:**
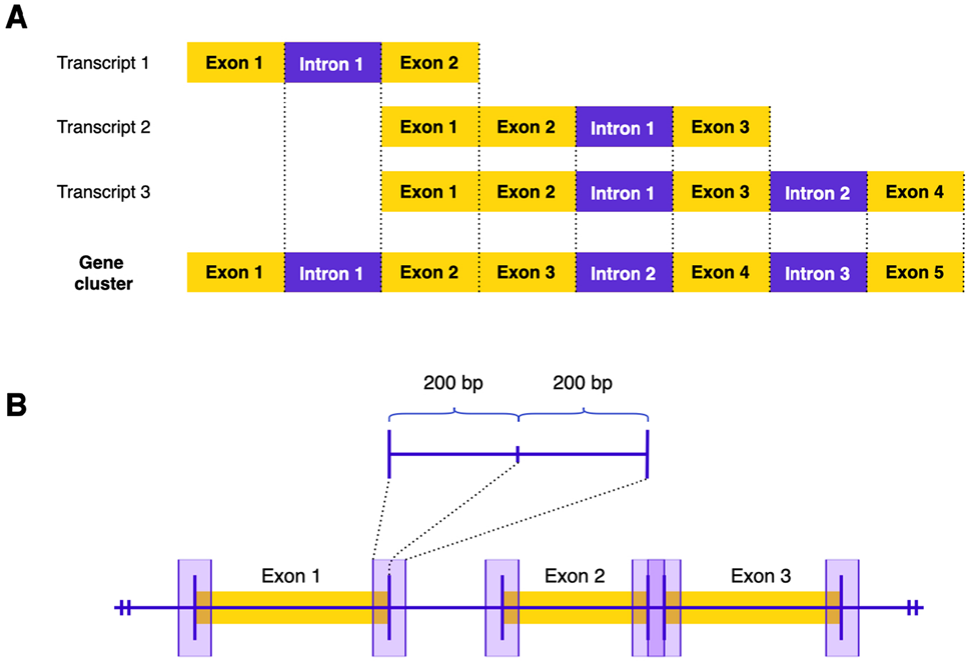
Redefinition of exons and exon flanks. (**A**) Overlapping transcript variants of a gene are collapsed and numbered in the flattened gene cluster following the strategy of Anders et al.^35^. Based on the read counts annotated to such redefined exons, DEXSeq compares the normalized exon usage between a tissue pair and determines differential exon usage (DEU) events. (**B**) Differential histone marks (DHMs) were detected by the tool MAnorm and annotated to exon borders (exon flanking regions), which were defined as the 200-bp regions around exon-intron junctions. These are flattened exons that are redefined following the scheme explained in (**A**).

The gene components of interest were annotated based on the NCBI human reference genome GRCh38. The GTF-formatted reference files were retrieved and flattened following Anders et al^35^. In the first step, we excluded overlapping genes that share at least one exon to avoid misannotation when mapping differential events to the reference genome. Instances of duplicated genes, genes spanning more than one genomic region and single-exon genes were discarded as well. Next, we extracted the unique exons and defined new gene clusters based on these exons using the HTSeq package^36^. If any two exons from different transcripts of the same gene were mapped to the same genomic region, they were rearranged by HTSeq and assigned to a new non-overlapping classification of exons that mapped to that region (Fig. 7A). These redefined exons and gene clusters were subjected to differential usage analysis by DEXSeq in the subsequent step^35,36^.

As introduced before, we assume a mechanistic foundation for epigenetically regulated splicing events that implies the crosstalk between splicing factors at a specific splice-site and the chromatin readers that are recruited in the vicinity. The effective range where such crosstalk is highly probable are termed “exon flanks” and were defined as 200-bp up- and downstream from an exon’s start or end sites (Fig. 7B) as was done in previous studies^17,19,37^. Data annotation for differentially modified histones was performed using the *intersect* command from the package BEDtools^38^. Note that the differential signals were annotated using the flattened exon model that is explained previously in this section.

### Differential analysis

#### Differential exon usage analysis

For the quantification of exon usage deregulation, the transcript and exon abundance in the polyA-plus RNA-seq alignment files were taken from the ENCODE database in BAM format. These BAM files were sorted lexicographically and converted to SAM format via SAMtools^39^. Using HTSeq, we obtained the read counts for flattened exons in each replicate of a sample from SAM files and used those as input for DEU analysis with the Bioconductor package DEXSeq^35^ for all possible pairs of samples between the 19 epigenomes.

In a pairwise comparison and for each exon, DEXSeq returns a statistic for differential usage and an FDR-adjusted p-value ($p_{FDR}$). The threshold of 0.05 was used to define significantly differentially expressed exons. Since we focused on the impact of DHMs on alternating splicing activity, we excluded the first exon of any transcript from the DEXSeq results, assuming that these are cases of alternate promoters where transcriptional regulatory effects of the investigated histone marks are more dominant^25^.

#### Differential histone modification analysis

As materials for the analysis, we procured the GRCh38-assembled and BAM-formatted alignment files and the BED-formatted replicated or pseudo-replicated peak files from histone ChIP-seq analysis for the five mentioned histone marks H3K27ac, H3K27me3, H3K36me3, H3K4me3 and H3K9me3. If multiple alignment files or peak files exist for a specific histone type and epigenome, they were merged using *merge* commands from SAMtools or BEDtools, respectively. To account for potential technical noise in the data and identify differentially modified histone regions, we modeled the epigenomic read counts using regression analysis in a pairwise manner across all epigenomes with MAnorm^40^. MAnorm returns the log2 ratio of read density between two samples ($M-value$) and a $p_{FDR}$ which we subsequently mapped to the flanking regions of each exon in the reference genome. The criteria for a flank to be differentially modified were $p_{FDR} \le 0.05$ as well as $|M-value| \ge 1$.

#### Multiple-comparison correction

The results from the pairwise comparisons across 19 cell types needed to be subjected to a multiple-testing correction to avoid an accumulation of false positives. This correction was implemented in the following manner: First, we computed the frequency of an exon having significant differential usage ($p_{FDR} \le 0.0001$) in one or more of the 171 pairwise comparisons across 19 tissues. As revealed by the cumulative distribution (Supplementary Fig. S1A in Supplementary Materials), about 95% of the respective individual exons have DEUs in only 1–25 comparisons. Those exons were labeled as “more tissue specific” due to their non-ubiquitous occurrences. For all following analyses, we only considered the set of genes containing such exons. For this restricted set of genes, we performed a “pooled” DEXSeq analysis using the full collection of samples belonging to all 19 selected cell types. This analysis reports all exons that are differentially used in at least one sample with respect to all other samples, as opposed to the previous pairwise DEXseq analysis. Performing this pooled analysis with DEXSeq on all exons for 171 pairwise comparisons would have been computationally prohibitive as observed in a preliminary test for a small subset of the data. Based on this integrated analysis, we identified all individual exons showing “pooled” differential usage with $p_{FDR} \le 0.05$ and filtered the results of the pairwise comparisons by keeping only these “overall significant” DEU exons. In the final dataset, we retained their DEU values from the pairwise comparisons, while setting the true values of non-significant exons to zero.

A multiple testing correction was likewise applied to the differentially abundant histone peaks that had been annotated to the exon flank regions of AS genes. For each region and each pairwise analysis, we retained the peak with the highest significance annotated to that region and performed an FDR correction on the results from all possible pairs. As significant DHM events, only those peaks with $p_{FDR} \le 0.05$ were retained.

### Identification and analysis of genes with strong DEU and DHM association

#### Overall and genewise co-occurrence of DEU and DHM

Previous work suggested that alteration of histone modifications contributes mechanistically to alternative splicing^6–11^. Hence, we first identified those exons where both types of rewiring events coincide. The frequency of such DEU-DHM co-occurrences was quantified by odds ratio (*OR*) as defined in Eq. (1).

1 $$\begin{aligned} OR = \frac{DEU\ \& \ DHM\ \times \ \lnot DEU\ \& \ \lnot DHM}{DEU\ \& \ \lnot DHM\ \times \ \lnot DEU\ \& \ DHM}, \end{aligned}$$

where $DEU\ \& \ DHM$ refers to the number of exons where both types of differential events were detected and $\lnot DEU\ \& \ \lnot DHM$ where none of the event types occurred. Exons with $DEU\ \& \lnot DHM$ or $\lnot DEU\ \& \ DHM$ were identified with either DEU or DHM events, respectively. An *OR* greater than 1 indicates a higher odd of occurrence for DEU in the presence of DHM, while *OR*s of 1 and less than 1 reflect that DEUs are either unaffected by DHMs or even underrepresented, respectively^41^. To determine the significance of these *OR*s, the p-values from Fisher Exact Tests (FET) ($p_{FET}$) were also computed and adjusted across all accounted exons.

For each type of histone modification, we first used a contingency table to categorize all exons based on their $DEU\ \& \ DHM$ overlaps to compute the *OR* and $p_{FET}$ significance for the set of genes where this hPTM type occurred (Table 1) and consider this as a “global” *OR* analysis. Second, we performed the analysis separately for all individual genes by means of computing gene-wise *OR*s and their statistical significance. The genes with strong evidence for a nonrandom association between epigenetic marks and splicing activity were defined by $p_{FET}$ $\le$ 0.05 and $OR\ge 1$ (Table S5 in Supplementary Materials). Finally, we performed the same analysis separately for all subgroups of genes annotated to separate biological process terms in the second or third hierarchy level of the Gene Ontology (GO). The point of this was to find out whether the co-occurrence of DEU and DHM was enriched or depleted in certain biological processes.

#### Combined differential expression analysis

Our next objective was to associate differential epigenetic profiles to exon rewiring of individual genes. For each individual gene and each pairwise comparison of epigenomes, we calculated the Pearson correlation between the DEXSeq-generated DEU values for all its exons and the respective $M-value$s computed by MAnorm mapped to their flanking regions (Fig. 7B). To enhance the contrast between differential and non-differential features, all DEU and DHM values with non-adjusted p-value > 0.05 were set to zero before computing the correlations. The top 5% of genes having the highest FDR-adjusted correlation of all genes between DEU and DHM (Fig. 3A) are referred to as “epispliced genes” in our study. Figure 1 provides an overview of the entire analysis.

We found many instances for *epispliced* genes where only one or a few exons show DEU-DHM overlaps and all other exons are annotated either to have only DEU or DHM events or even none of them. For our analysis, where we associate differential splicing with differential histone modifications, those rare DEU-DHM exons should be considered as true signals and should not be mistaken as outliers. Figure S1C in Supplementary Materials compares results from both Spearman rank correlation and from Pearson correlation. In most cases, Spearman correlation gave slightly smaller coefficients than Pearson correlation and identified approximately half as many *epispliced* genes. However, 88% of the *epispliced* genes identified by Spearman were also identified by Pearson and all downstream analyses showed the same trends.

#### Association between *epispliced* genes and human development

For each histone modification type, we counted how many *epispliced* genes or gene clusters (identified in any pairwise comparison involving this sample) are shared between two cell types. As a similarity measure of shared episplicing between two cell types, the Jaccard index (Eq.  2) was used:

2 $$\begin{aligned} J(E_1, E_2) = \frac{E_1 \cap E_2}{E_1 \cup E_2}, \end{aligned}$$

where $E_1$ and $E_2$ are the sets of *epispliced* genes identified for a pair of cells or tissues.

Additionally, we quantified how well the cell type labels matched the similarity of episplicing on the basis of adjusted Rand indices. For this, the epigenomes were first annotated based either on their potency (potency), the sample type retrieved from ENCODE database (sample type), the germ layer they originate from (origin) or the life stage to which they belong (life stage). Then, we defined pairwise distances between epigenomes by subtracting their Jaccard similarity index of shared *epispliced* genes from 100%. These distances were then used for hierarchical clustering of the epigenomes. Using *adj.rand.index()* function from the CRAN package *fossil*, the matching between the true labels and *epispliced* genes-based clusters was quantified.

Finally, all non-ubiquitous *epispliced* genes (identified in 1–25 pairwise comparisons) collected for each histone mark were subjected to GO term enrichment analysis according to the biological process hierarchy of the PANTHER classification system^42^. The background gene set used for computing enrichment comprises all genes having either DEU or DHM events at their exon flank regions. GO term enrichment analysis was performed using the Bioconductor package *clusterProfiler* with a cutoff $p_{FDR} \le 0.05$ for significant enrichment level^43^ . Enriched GO terms were sorted in decreasing order of fold enrichment.

## Supplementary information

Supplementary Information.

## Data Availability

RNA-seq and ChIP-seq data used in this study are parts of the Roadmap Epigenomics Project^21^ and are available on ENCODE database^34^ at https://www.encodeproject.org/. The detailed descriptions on biosamples used for the analysis can be found in Supplementary Tables S2–S4. All analysis code and additional data supporting the study are accessible via https://github.com/dhtt/ENCODE_episplicing.git.

## References

[1] Mironov AA, Fickett JW, Gelfand MS (1999). Frequent alternative splicing of human genes. Genome Res..

[2] Koscielny G (2009). ASTD: The alternative splicing and transcript diversity database. Genomics.

[3] Nilsen TW, Graveley BR (2010). Expansion of the eukaryotic proteome by alternative splicing. Nature.

[4] Wang ET (2008). Alternative isoform regulation in human tissue transcriptomes. Nature.

[5] Djebali S (2012). Landscape of transcription in human cells. Nature.

[6] Allo M (2010). Chromatin and alternative splicing. Cold Spring Harb. Symp. Quant. Biol..

[7] Luco RF, Allo M, Schor IE, Kornblihtt AR, Misteli T (2011). Epigenetics in alternative pre-mRNA splicing. Cell.

[8] Zhou HL, Luo G, Wise JA, Lou H (2014). Regulation of alternative splicing by local histone modifications: Potential roles for RNA-guided mechanisms. Nucleic Acids Res..

[9] de Klerk E, t Hoen PA (2015). Alternative mRNA transcription, processing, and translation: Insights from RNA sequencing. Trends Genet..

[10] Adami G, Babiss LE (1991). DNA template effect on RNA splicing: Two copies of the same gene in the same nucleus are processed differently. EMBO J..

[11] Luco RF (2010). Regulation of alternative splicing by histone modifications. Science.

[12] Segelle A (2022). Histone marks regulate the epithelial-to-mesenchymal transition via alternative splicing. Cell Rep..

[13] Kalsotra A, Cooper TA (2011). Functional consequences of developmentally regulated alternative splicing. Nat. Rev. Genet..

[14] Baralle FE, Giudice J (2017). Alternative splicing as a regulator of development and tissue identity. Nat. Rev. Mol. Cell Biol..

[15] Bebee TW (2015). The splicing regulators ESRP1 and ESRP2 direct an epithelial splicing program essential for mammalian development. Elife..

[16] Weyn-Vanhentenryck SM (2018). Precise temporal regulation of alternative splicing during neural development. Nat. Commun..

[17] Hu Q, Greene CS, Heller EA (2020). Specific histone modifications associate with alternative exon selection during mammalian development. Nucleic Acids Res..

[18] Liu H, Jin T, Guan J, Zhou S (2014). Histone modifications involved in cassette exon inclusions: A quantitative and interpretable analysis. BMC Genom..

[19] Zhou Y, Lu Y, Tian W (2012). Epigenetic features are significantly associated with alternative splicing. BMC Genom..

[20] Lee D, Zhang J, Liu J, Gerstein M (2020). Epigenome-based splicing prediction using a recurrent neural network. PLoS Comput. Biol..

[21] Roadmap Epigenomics C (2015). Integrative analysis of 111 reference human epigenomes. Nature.

[22] Podlaha O, De S, Gonen M, Michor F (2014). Histone modifications are associated with transcript isoform diversity in normal and cancer cells. PLoS Comput. Biol..

[23] Zheng Z, Wei X, Hildebrandt A, Schmidt B (2016). A computational method for studying the relation between alternative splicing and DNA methylation. Nucleic Acids Res..

[24] Enroth S, Bornelov S, Wadelius C, Komorowski J (2012). Combinations of histone modifications mark exon inclusion levels. PLoS One.

[25] Pal S (2011). Alternative transcription exceeds alternative splicing in generating the transcriptome diversity of cerebellar development. Genome Res..

[26] Draaken M (2012). Involvement of the WNT and FGF signaling pathways in non-isolated anorectal malformations: sequencing analysis of WNT3A, WNT5A, WNT11, DACT1, FGF10, FGFR2 and the T gene. Int. J. Mol. Med..

[27] Ke X-S (2010). Global profiling of histone and DNA methylation reveals epigenetic-based regulation of gene expression during epithelial to mesenchymal transition in prostate cells. BMC Genom..

[28] Jacob AG, Smith CW (2017). Intron retention as a component of regulated gene expression programs. Hum. Genet..

[29] Creyghton MP (2010). Histone H3K27ac separates active from poised enhancers and predicts developmental state. Proc. Natl. Acad. Sci..

[30] Benayoun B (2014). H3K4me3 breadth is linked to cell identity and transcriptional consistency. Cell.

[31] Burney MJ (2013). An epigenetic signature of developmental potential in neural stem cells and early neurons. Stem Cells.

[32] Mikkelsen TS (2007). Genome-wide maps of chromatin state in pluripotent and lineage-committed cells. Nature.

[33] Huang H, Weng H, Chen J (2020). The biogenesis and precise control of RNA m6A methylation. Trends Genet..

[34] Davis CA (2018). The Encyclopedia of DNA elements (ENCODE): Data portal update. Nucleic Acids Res..

[35] Anders S, Reyes A, Huber W (2012). Detecting differential usage of exons from RNA-seq data. Genome Res..

[36] Anders S, Pyl PT, Huber W (2015). HTSeq-a Python framework to work with high-throughput sequencing data. Bioinformatics.

[37] Hu Q, Kim EJ, Feng J, Grant GR, Heller EA (2017). Histone posttranslational modifications predict specific alternative exon subtypes in mammalian brain. PLoS Comput. Biol..

[38] Quinlan AR, Hall IM (2010). BEDTools: A flexible suite of utilities for comparing genomic features. Bioinformatics.

[39] Li H (2009). The sequence alignment/map format and SAMtools. Bioinformatics.

[40] Shao Z, Zhang Y, Yuan GC, Orkin SH, Waxman DJ (2012). MAnorm: A robust model for quantitative comparison of ChIP-Seq data sets. Genome Biol..

[41] Szumilas M (2010). Explaining odds ratios. J. Can. Acad. Child Adolesc. Psychiatry.

[42] Mi H, Muruganujan A, Casagrande JT, Thomas PD (2013). Large-scale gene function analysis with the PANTHER classification system. Nat. Protoc..

[43] Wu T (2021). clusterProfiler 4.0: A universal enrichment tool for interpreting omics data. Innovation.

