# Supplementary material for: Differential exon usage of developmental genes is associated with deregulated epigenetic marks
Source: Sci Rep. 2023 Jul 28;13:12256. doi: 10.1038/s41598-023-38879-z (PMC10382575; doi:10.1038/s41598-023-38879-z)
Supplement: Supplementary file 1 — Supplementary Information. [file 41598_2023_38879_MOESM1_ESM.pdf]

Supplementary Information
for
Differential Exon Usage of Developmental Genes is Associated with Deregulated Epigenetic Marks

Hoang Thu Trang Do[1], Siba Shanak[2], Ahmad Barghash[3], and Volkhard Helms[1,*]

[1]Center for Bioinformatics, Saarland University, Saarbrücken, Germany
[2]Department of Biology and Biotechnology, Arab American University, Jenin, Palestine
[3]Department of Computer Science, German Jordanian University, Amman, Jordan
[*]volkhard.helms@bioinformatik.uni-saarland.de

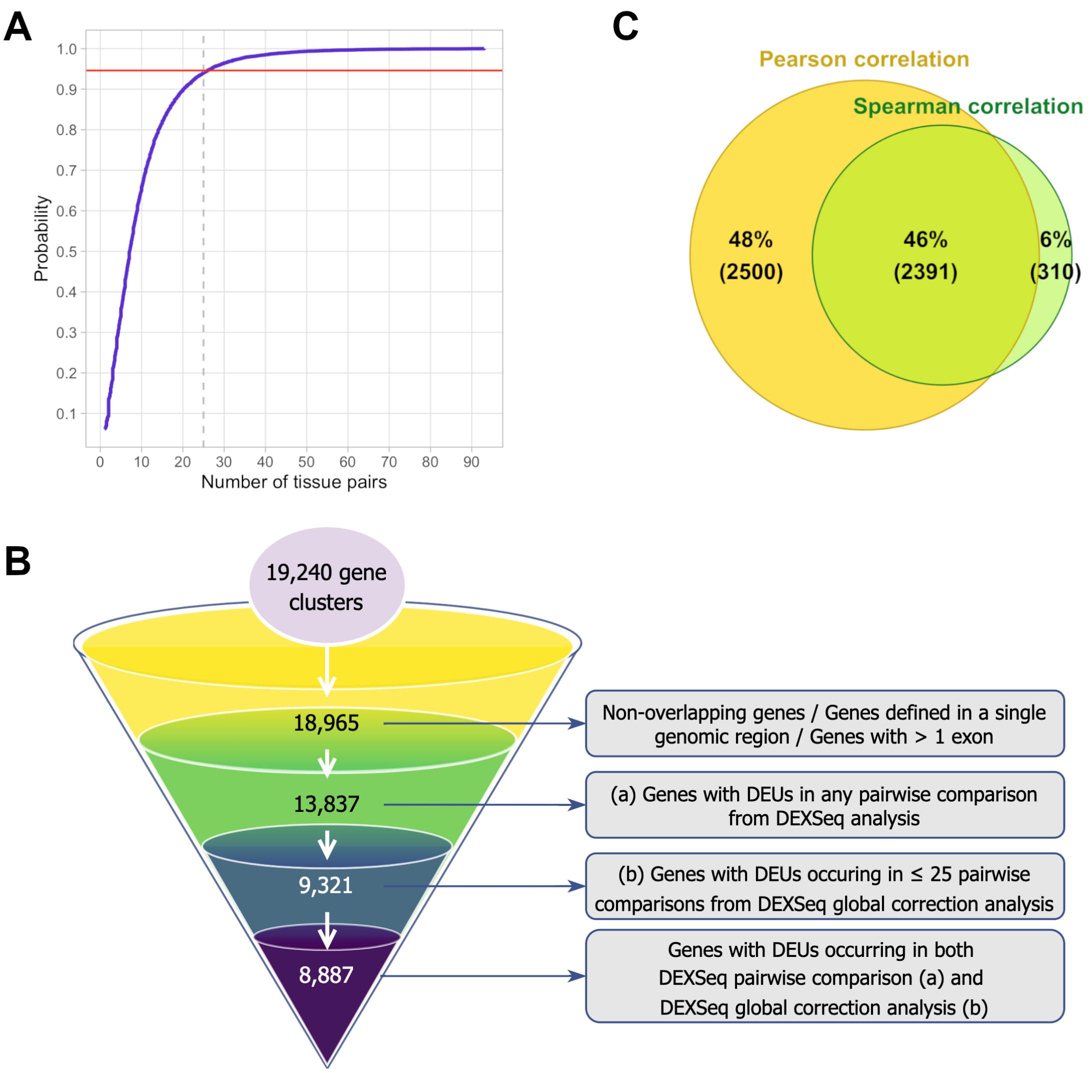

Supplementary Figure S1: Preliminary results for defining the analysis method. (A) Cumulative distribution for the number of tissue pairs in which a differentially used exon is identified. Approximately 95% of the differentially used exons were detected in fewer than 25 pairwise comparisons across 19 tissues (171 pairs in total). These non-ubiquitous, differentially used exons belong to 9,321 genes that were considered for the identification of epispliced genes. (B) Diagram demonstrating the genes selection process for the correlation analysis between differential exon usage (DEU) and differential histone modification (DHM). From the initial 19,240 flattened gene clusters, 275 clusters of genes partially overlapping with each other, spanning more than one genomic region or containing only a single exon were first excluded. The remaining genes were used for pairwise DEU analysis with DEXSeq, resulting in the set of 13,837 genes containing at least one DEU event in any pairwise comparison (a). Using only 9,321 genes with non-ubiquitous DEUs detected in (A), a global DEXSeq analysis was performed to compare all samples against each other simultaneously for multiple testings correction purpose (b). The set of genes with DEUs occuring in both the separate pairwise comparison (a) and the global correction (b) consists of 8,887 genes and was subjected to the main correlation analysis of the study. (C) Venn diagram showing the overlap between the sets of epispliced genes from the analysis with Pearson correlation and Spearman rank correlation.

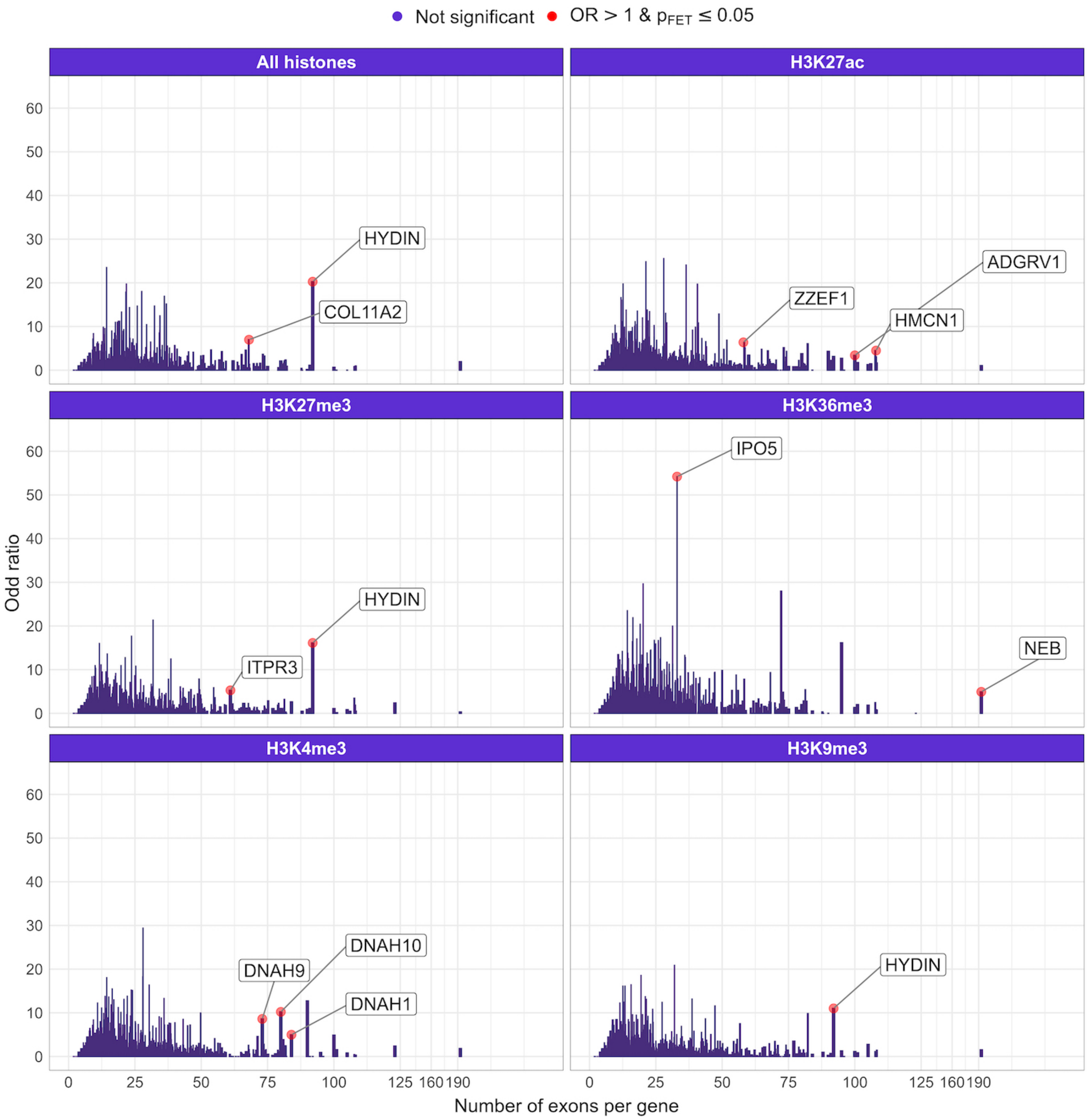

Supplementary Figure S2: Genewise odds ratio with respect to number of exons. The genes with $OR > 1$ and FDR-adjusted $p - value \leq 0.05$ from Fisher Exact Test are highlighted in red. The odds ratios distribution with respect to the number of exons are shown for 5 histone marks H3K27ac, H3K27me3, H3K36me3, H3K4me3 and H3K9me3, as well as for any detected differential histone peaks regardless of histone modification type (All histones).

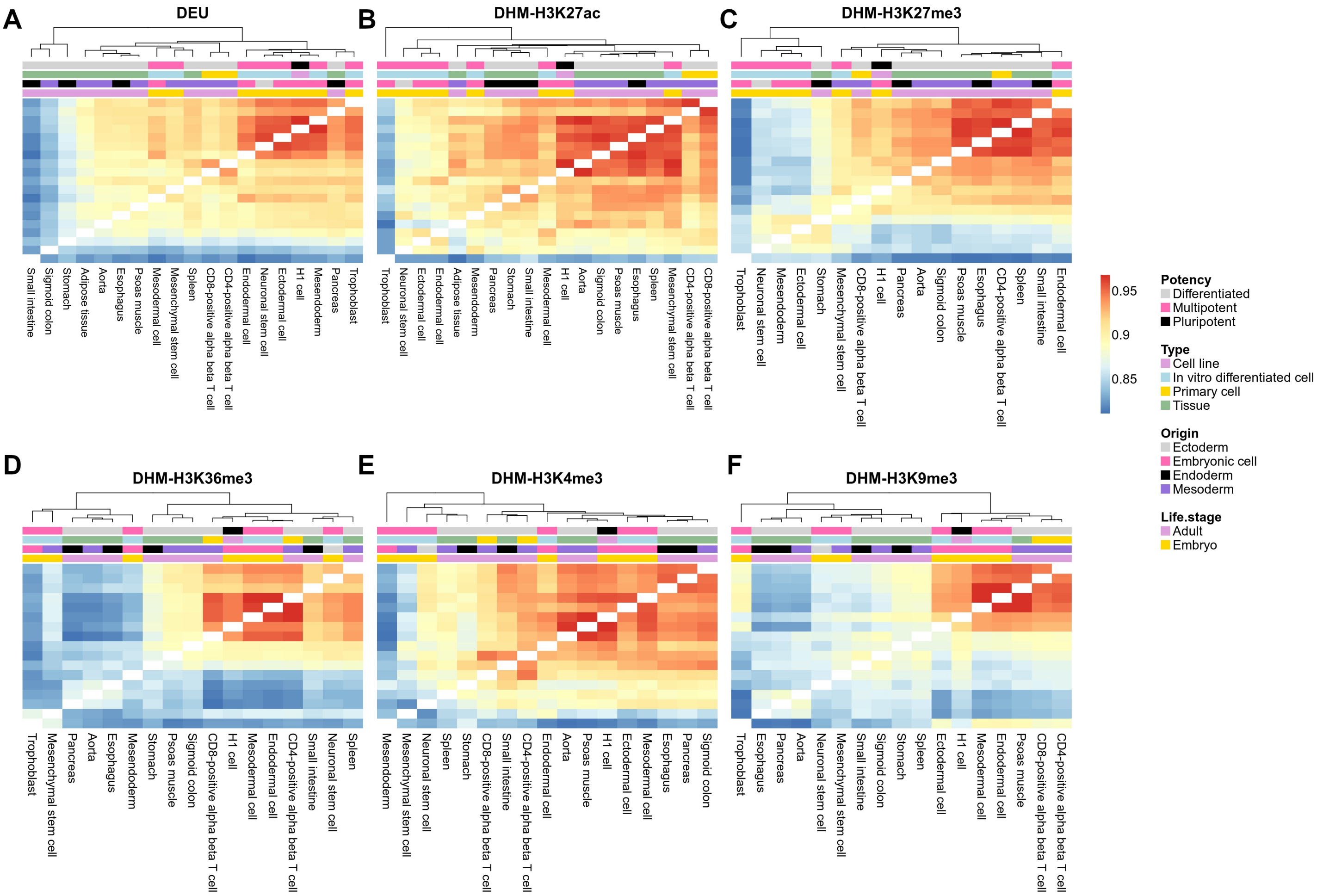

Supplementary Figure S3: Heatmaps representing hierarchical clustering of studied tissues based on genes with differential exon usage (DEU) or differential histone modification (DHM). The pairwise distance across a total of 19 tissues were computed using the set of genes with differential features identified from DEXSeq DEU analysis (A) and MAnorm DHM analysis for H3K27ac, H3K27me3, H3K36me, H3K4me3 and H3K9me3 (B-F). For each differential feature, the tissue distance was measured by Jaccard index, defined as the ratio between the number of mutually affected genes and the total number of affected genes in the union sets of two cell types. All heatmaps use the same color scale ranging from 0.86 to the highest Jaccard index across all tissue pairs and for different differential features. Investigated epigenomes were annotated on the top by their differentiation potency, type of sample, germ layer origin and the life stage when their samples were taken.

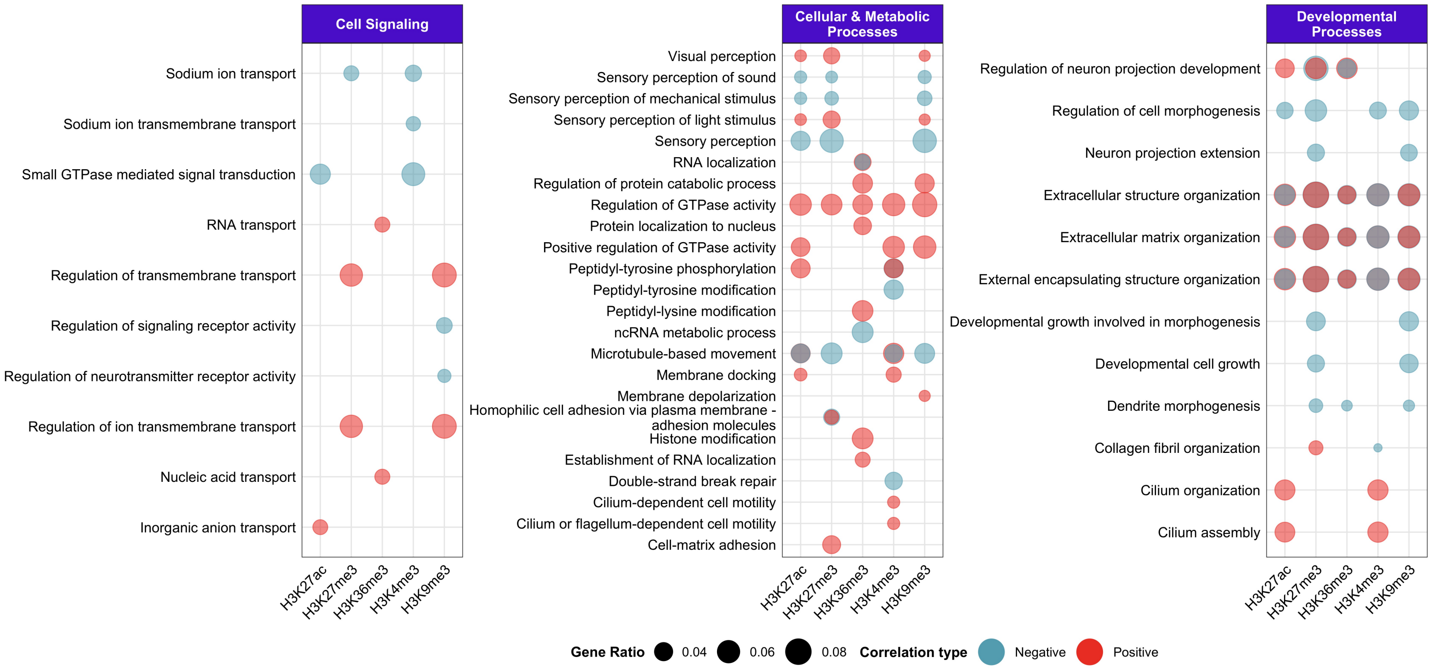

Supplementary Figure S4: Gene ontology (GO) enrichment analysis for biological functions of non-ubiquitous epispliced genes for each histone type. The top enriched GO terms (FDR-adjusted $p-value \leq 0.05$) annotated to epispliced genes that were correlated either with H3K27ac, H3K27me3, H3K36me, H3K4me3 or with H3K9me3 differential histone modifications (DHM) were sorted in decreasing order of significance and of mutual functions between the histone marks. The terms annotated to the epispliced genes with positive correlation between differential exon usage (DEU) are represented by red and with negative correlation by blue. All GO terms are grouped into three main categories, namely cell signaling (A), cellular and metabolic processes (B) and developmental processes (C). In the enrichment analysis, the respective epispliced gene sets were compared against the background set of all human genes having either DEU or DHM events at the exon boundaries.

Epigenome	Potency	Type	Origin	Life stage
**Adipose tissue**	Differentiated	Tissue	Mesoderm	Adult
**Aorta**	Differentiated	Tissue	Mesoderm	Adult
**CD4-positive alpha beta T cell**	Differentiated	Primary cell	Mesoderm	Adult
**CD8-positive alpha beta T cell**	Differentiated	Primary cell	Mesoderm	Adult
**Ectodermal cell**	Multipotent	In vitro differentiated cell	Embryonic cell	Embryo
**Endodermal cell**	Multipotent	In vitro differentiated cell	Embryonic cell	Embryo
**Esophagus**	Differentiated	Tissue	Endoderm	Adult
**H1 cell**	Pluripotent	Cell line	Embryonic cell	Embryo
**Mesenchymal stem cell**	Multipotent	In vitro differentiated cell	Mesoderm	Embryo
**Mesendoderm**	Multipotent	In vitro differentiated cell	Embryonic cell	Embryo
**Mesodermal cell**	Multipotent	In vitro differentiated cell	Embryonic cell	Embryo
**Neuronalstem cell**	Multipotent	In vitro differentiated cell	Ectoderm	Embryo
**Pancreas**	Differentiated	Tissue	Endoderm	Adult
**Psoas muscle**	Differentiated	Tissue	Mesoderm	Adult
**Sigmoid colon**	Differentiated	Tissue	Mesoderm	Adult
**Small intestine**	Differentiated	Tissue	Endoderm	Adult
**Spleen**	Differentiated	Tissue	Mesoderm	Adult
**Stomach**	Differentiated	Tissue	Endoderm	Adult
**Trophoblast cell**	Multipotent	In vitro differentiated cell	Embryonic cell	Embryo

Supplementary Table S1: List of tissues and cell types retrieved from the Human Epigenome Atlas with annotated potency, sample type, origin and life stage.

Biosample term name	File accession	Experiment accession	File format	Output type	Biosample type	Biological replicate(s)	Technical replicate(s)
adipose tissue	ENCFF717MIN	ENCSR686JJB	bam	alignments	tissue	1	1_1
adipose tissue	ENCFF491UPQ	ENCSR741QEH	bam	alignments	tissue	1	1_2
aorta	ENCFF081DYZ	ENCSR763NOO	bam	alignments	tissue	1	1_2
aorta	ENCFF864QLZ	ENCSR995BHD	bam	alignments	tissue	1	1_1
CD4-positive, alpha-beta T cell	ENCFF597BAH	ENCSR545MEZ	bam	alignments	primary cell	1	1_1
CD4-positive, alpha-beta T cell	ENCFF208TSZ	ENCSR463JBR	bam	alignments	primary cell	1	1_1
CD8-positive, alpha-beta T cell	ENCFF746WLK	ENCSR944FLL	bam	alignments	primary cell	1	1_1
CD8-positive, alpha-beta T cell	ENCFF716ABE	ENCSR861QKF	bam	alignments	primary cell	1	1_1
ectodermal cell	ENCFF985MTW	ENCSR872LTT	bam	alignments	in vitro differentiated cells	1	1_1
ectodermal cell	ENCFF148VCG	ENCSR872LTT	bam	alignments	in vitro differentiated cells	1	1_2
ectodermal cell	ENCFF549FQD	ENCSR382XJF	bam	alignments	in vitro differentiated cells	1	1_3
ectodermal cell	ENCFF370OWM	ENCSR851BRK	bam	alignments	in vitro differentiated cells	1	1_3
ectodermal cell	ENCFF686CNC	ENCSR593AMV	bam	alignments	in vitro differentiated cells	1	1_1
ectodermal cell	ENCFF154POQ	ENCSR593AMV	bam	alignments	in vitro differentiated cells	1	1_2
endodermal cell	ENCFF489LAR	ENCSR002CTR	bam	alignments	in vitro differentiated cells	1	1_3
endodermal cell	ENCFF093GMX	ENCSR472PBS	bam	alignments	in vitro differentiated cells	1	1_3
esophagus	ENCFF441FVJ	ENCSR993QGR	bam	alignments	tissue	1	1_1
esophagus	ENCFF251YUZ	ENCSR102TQN	bam	alignments	tissue	1	1_2
H1	ENCFF740OFO	ENCSR043RSE	bam	alignments	cell line	1	1_1
H1	ENCFF365DFR	ENCSR670WQY	bam	alignments	cell line	1	1_1
mesenchymal stem cell	ENCFF011JJG	ENCSR663WGC	bam	alignments	in vitro differentiated cells	1	1_1
mesenchymal stem cell	ENCFF317PIS	ENCSR275ZLF	bam	alignments	in vitro differentiated cells	1	1_1
mesendoderm	ENCFF738KXI	ENCSR700BEW	bam	alignments	in vitro differentiated cells	1	1_1
mesendoderm	ENCFF028PIJ	ENCSR976JGI	bam	alignments	in vitro differentiated cells	1	1_1
mesodermal cell	ENCFF107QNN	ENCSR500UOD	bam	alignments	in vitro differentiated cells	1	1_3
mesodermal cell	ENCFF091OZL	ENCSR433GXV	bam	alignments	in vitro differentiated cells	1	1_3
neuronal stem cell	ENCFF359YOQ	ENCSR977XUX	bam	alignments	in vitro differentiated cells	1	1_1
neuronal stem cell	ENCFF905ZXT	ENCSR572EET	bam	alignments	in vitro differentiated cells	1	1_1
pancreas	ENCFF199EFU	ENCSR629VMZ	bam	alignments	tissue	1	1_1
pancreas	ENCFF085TWC	ENCSR571BML	bam	alignments	tissue	1	1_1
psoas muscle	ENCFF677DKS	ENCSR502OTI	bam	alignments	tissue	1	1_1
psoas muscle	ENCFF738TTD	ENCSR843HXR	bam	alignments	tissue	1	1_1
sigmoid colon	ENCFF588TQX	ENCSR825GWD	bam	alignments	tissue	1	1_12
sigmoid colon	ENCFF050JVY	ENCSR999ZCI	bam	alignments	tissue	1	1_1
small intestine	ENCFF584GAJ	ENCSR039ICU	bam	alignments	tissue	1	1_1
small intestine	ENCFF894PUN	ENCSR719HRO	bam	alignments	tissue	1	1_1
spleen	ENCFF717MVQ	ENCSR510PSL	bam	alignments	tissue	1	1_2
spleen	ENCFF918SPI	ENCSR910QOX	bam	alignments	tissue	1	1_2
stomach	ENCFF268XDH	ENCSR721HDG	bam	alignments	tissue	1	1_1
stomach	ENCFF056CIU	ENCSR980UEY	bam	alignments	tissue	1	1_1
trophoblast cell	ENCFF137MXA	ENCSR762CJN	bam	alignments	in vitro differentiated cells	1	1_1
trophoblast cell	ENCFF677DAD	ENCSR762CJN	bam	alignments	in vitro differentiated cells	2	2_1

Biosample term name	File accession	Experiment accession	File format	Output type	Biosample type	Biological replicate(s)	Technical replicate(s)

Supplementary Table S2: Metadata for the retrieved Human Epigenome Atlas poly-A plus RNA-seq. For each biosample, the associated information including the file accession number, experiment accession number, file format, sample types and replicates are listed. All files used the Human Reference Genome GRCh38 for assembly.

Biosample term name	File accession	Experiment accession	File format	Output type	Experiment target	Biological replicate(s)	Technical replicate(s)
adipose tissue	ENCFF861HMY	ENCSR082SHT	bam	alignments	H3K27ac-human	1	1_1
aorta	ENCFF434DCE	ENCSR322TJD	bam	alignments	H3K27ac-human	1	1_2
aorta	ENCFF265HDL	ENCSR519CFV	bam	alignments	H3K27ac-human	2	2_1
CD4-positive, alpha-beta T cell	ENCFF082MIE	ENCSR561KOM	bam	alignments	H3K27ac-human	1	1_2
CD8-positive, alpha-beta T cell	ENCFF449YKU	ENCSR007HLH	bam	alignments	H3K27ac-human	1	1_2
ectodermal cell	ENCFF728FKI	ENCSR747HAM	bam	alignments	H3K27ac-human	1	1_2
ectodermal cell	ENCFF537TER	ENCSR747HAM	bam	alignments	H3K27ac-human	2	2_2
endodermal cell	ENCFF898KBV	ENCSR200ETW	bam	alignments	H3K27ac-human	1	1_2
endodermal cell	ENCFF668YUL	ENCSR200ETW	bam	alignments	H3K27ac-human	2	2_2
esophagus	ENCFF303TFY	ENCSR679OVD	bam	alignments	H3K27ac-human	1	1_1
esophagus	ENCFF166HVM	ENCSR645SYH	bam	alignments	H3K27ac-human	1	1_1
H1	ENCFF948UXT	ENCSR880SUY	bam	alignments	H3K27ac-human	2	2_1
H1	ENCFF663SAM	ENCSR880SUY	bam	alignments	H3K27ac-human	1	1_2
mesenchymal stem cell	ENCFF262VKZ	ENCSR013KEC	bam	alignments	H3K27ac-human	1	1_1
mesenchymal stem cell	ENCFF615PUT	ENCSR013KEC	bam	alignments	H3K27ac-human	2	2_1
mesendoderm	ENCFF710LJW	ENCSR473PNT	bam	alignments	H3K27ac-human	1	1_1
mesendoderm	ENCFF535JGD	ENCSR473PNT	bam	alignments	H3K27ac-human	2	2_1
mesodermal cell	ENCFF114JZY	ENCSR931WLE	bam	alignments	H3K27ac-human	1	1_2
mesodermal cell	ENCFF175GQI	ENCSR931WLE	bam	alignments	H3K27ac-human	2	2_2
neuronal stem cell	ENCFF063LDJ	ENCSR799SRL	bam	alignments	H3K27ac-human	1	1_1
neuronal stem cell	ENCFF402MPW	ENCSR799SRL	bam	alignments	H3K27ac-human	2	2_1
neuronal stem cell	ENCFF263UOB	ENCSR799SRL	bam	alignments	H3K27ac-human	3	3_1
pancreas	ENCFF015LEU	ENCSR402HFW	bam	alignments	H3K27ac-human	1	1_1
pancreas	ENCFF516INT	ENCSR612BWE	bam	alignments	H3K27ac-human	1	1_1
psoas muscle	ENCFF295IFC	ENCSR250NHD	bam	alignments	H3K27ac-human	1	1_1
psoas muscle	ENCFF755AFZ	ENCSR367WYJ	bam	alignments	H3K27ac-human	1	1_1, 1_2
psoas muscle	ENCFF305JAV	ENCSR791ISZ	bam	alignments	H3K27ac-human	2	2_1
sigmoid colon	ENCFF118ACN	ENCSR213SMK	bam	alignments	H3K27ac-human	1	1_1
sigmoid colon	ENCFF342MZW	ENCSR561YSH	bam	alignments	H3K27ac-human	1	1_1
small intestine	ENCFF429QHU	ENCSR543CPW	bam	alignments	H3K27ac-human	1	1_3
small intestine	ENCFF410WCE	ENCSR655XLM	bam	alignments	H3K27ac-human	1	1_1
small intestine	ENCFF915JKN	ENCSR454VRA	bam	alignments	H3K27ac-human	1	1_1
small intestine	ENCFF466VBP	ENCSR892XFG	bam	alignments	H3K27ac-human	1	1_2
spleen	ENCFF825ZUF	ENCSR170MAJ	bam	alignments	H3K27ac-human	1	1_1
spleen	ENCFF628GTH	ENCSR170MAJ	bam	alignments	H3K27ac-human	1	1_1
spleen	ENCFF850VCJ	ENCSR235ZBF	bam	alignments	H3K27ac-human	1	1_2
spleen	ENCFF500MVV	ENCSR235ZBF	bam	alignments	H3K27ac-human	1	1_2
spleen	ENCFF062ZGG	ENCSR086XCT	bam	alignments	H3K27ac-human	1	1_1
spleen	ENCFF626HSF	ENCSR086XCT	bam	alignments	H3K27ac-human	1	1_1
stomach	ENCFF735KUK	ENCSR001SHB	bam	alignments	H3K27ac-human	1	1_2
stomach	ENCFF972FYM	ENCSR437QMD	bam	alignments	H3K27ac-human	1	1_1, 1_2
stomach	ENCFF081CDJ	ENCSR743DDX	bam	alignments	H3K27ac-human	1	1_2
stomach	ENCFF805CCB	ENCSR582UTE	bam	alignments	H3K27ac-human	1	1_1
trophoblast cell	ENCFF233ZTK	ENCSR425PQI	bam	alignments	H3K27ac-human	1	1_1
trophoblast cell	ENCFF570NQD	ENCSR425PQI	bam	alignments	H3K27ac-human	2	2_1
trophoblast cell	ENCFF625RQZ	ENCSR425PQI	bam	alignments	H3K27ac-human	2	2_1

Biosample term name	File accession	Experiment accession	File format	Output type	Experiment target	Biological replicate(s)	Technical replicate(s)
aorta	ENCFF127BNG	ENCSR196PGM	bam	alignments	H3K27me3-human	1	1_1
aorta	ENCFF654BWT	ENCSR128VHV	bam	alignments	H3K27me3-human	1	1_2
CD4-positive, alpha-beta T cell	ENCFF971YVS	ENCSR043SBG	bam	alignments	H3K27me3-human	1	1_4
CD4-positive, alpha-beta T cell	ENCFF624YMM	ENCSR733QOZ	bam	alignments	H3K27me3-human	1	1_2
CD8-positive, alpha-beta T cell	ENCFF783NPA	ENCSR103GGR	bam	alignments	H3K27me3-human	1	1_4
ectodermal cell	ENCFF761AOT	ENCSR690GLT	bam	alignments	H3K27me3-human	1	1_2
endodermal cell	ENCFF465YTB	ENCSR273IYV	bam	alignments	H3K27me3-human	2	2_5
endodermal cell	ENCFF249NJY	ENCSR273IYV	bam	alignments	H3K27me3-human	1	1_5
esophagus	ENCFF096RVZ	ENCSR641RQV	bam	alignments	H3K27me3-human	1	1_2
esophagus	ENCFF703GII	ENCSR088GXB	bam	alignments	H3K27me3-human	1	1_1
H1	ENCFF748KOZ	ENCSR186OBR	bam	alignments	H3K27me3-human	1	1_1
H1	ENCFF382GPJ	ENCSR186OBR	bam	alignments	H3K27me3-human	2	2_1
H1	ENCFF596SHE	ENCSR928HYM	bam	alignments	H3K27me3-human	1	1_2
H1	ENCFF830PVE	ENCSR928HYM	bam	alignments	H3K27me3-human	2	2_1
H1	ENCFF310SBN	ENCSR216OGD	bam	alignments	H3K27me3-human	2	2_1
H1	ENCFF083QQZ	ENCSR216OGD	bam	alignments	H3K27me3-human	1	1_1
mesenchymal stem cell	ENCFF356HLU	ENCSR832JVP	bam	alignments	H3K27me3-human	1	1_1
mesenchymal stem cell	ENCFF455IUR	ENCSR832JVP	bam	alignments	H3K27me3-human	2	2_1
mesendoderm	ENCFF803MMH	ENCSR405AXO	bam	alignments	H3K27me3-human	1	1_1
mesendoderm	ENCFF244AUB	ENCSR405AXO	bam	alignments	H3K27me3-human	2	2_1
neuronal stem cell	ENCFF230ZHL	ENCSR550XZG	bam	alignments	H3K27me3-human	2	2_1
neuronal stem cell	ENCFF101MMK	ENCSR550XZG	bam	alignments	H3K27me3-human	1	1_1
neuronal stem cell	ENCFF194JFG	ENCSR692CTK	bam	alignments	H3K27me3-human	1	1_1
neuronal stem cell	ENCFF055OMW	ENCSR692CTK	bam	alignments	H3K27me3-human	2	2_1
neuronal stem cell	ENCFF781FVD	ENCSR694LBI	bam	alignments	H3K27me3-human	1	1_1
neuronal stem cell	ENCFF591ZEN	ENCSR694LBI	bam	alignments	H3K27me3-human	2	2_1
pancreas	ENCFF530LYL	ENCSR186QKH	bam	alignments	H3K27me3-human	1	1_1
pancreas	ENCFF987RZA	ENCSR486NDF	bam	alignments	H3K27me3-human	1	1_1, 1_2
psoas muscle	ENCFF096XQY	ENCSR720SAS	bam	alignments	H3K27me3-human	1	1_1
psoas muscle	ENCFF539ZGZ	ENCSR843KHS	bam	alignments	H3K27me3-human	1	1_2
sigmoid colon	ENCFF124DET	ENCSR042RIW	bam	alignments	H3K27me3-human	1	1_1
sigmoid colon	ENCFF607WIJ	ENCSR897TGR	bam	alignments	H3K27me3-human	1	1_1
small intestine	ENCFF557ZVM	ENCSR877PAS	bam	alignments	H3K27me3-human	1	1_2
small intestine	ENCFF816GHG	ENCSR340OPI	bam	alignments	H3K27me3-human	1	1_1
small intestine	ENCFF651ZOQ	ENCSR859EIX	bam	alignments	H3K27me3-human	1	1_2
spleen	ENCFF558UBX	ENCSR608FDQ	bam	alignments	H3K27me3-human	1	1_2
spleen	ENCFF061EVZ	ENCSR608FDQ	bam	alignments	H3K27me3-human	1	1_2
spleen	ENCFF708NMK	ENCSR408ONP	bam	alignments	H3K27me3-human	1	1_1
spleen	ENCFF191LZK	ENCSR408ONP	bam	alignments	H3K27me3-human	1	1_1
stomach	ENCFF192EMA	ENCSR527BFF	bam	alignments	H3K27me3-human	1	1_1
stomach	ENCFF582NZD	ENCSR354IST	bam	alignments	H3K27me3-human	1	1_2
trophoblast cell	ENCFF904JLV	ENCSR960CWQ	bam	alignments	H3K27me3-human	1	1_1
trophoblast cell	ENCFF303YNU	ENCSR960CWQ	bam	alignments	H3K27me3-human	2	2_1
trophoblast cell	ENCFF482DKF	ENCSR960CWQ	bam	alignments	H3K27me3-human	2	2_1
aorta	ENCFF168IPJ	ENCSR673JYT	bam	alignments	H3K36me3-human	1	1_1
aorta	ENCFF230YGV	ENCSR989AMI	bam	alignments	H3K36me3-human	1	1_1

Biosample term name	File accession	Experiment accession	File format	Output type	Experiment target	Biological replicate(s)	Technical replicate(s)
CD4-positive, alpha-beta T cell	ENCFF300XBB	ENCSR828WZG	bam	alignments	H3K36me3-human	1	1_4
CD4-positive, alpha-beta T cell	ENCFF151UEE	ENCSR774OKQ	bam	alignments	H3K36me3-human	1	1_3
CD8-positive, alpha-beta T cell	ENCFF814JIZ	ENCSR782NOO	bam	alignments	H3K36me3-human	1	1_3
endodermal cell	ENCFF778DVB	ENCSR677EZB	bam	alignments	H3K36me3-human	1	1_4
esophagus	ENCFF168XVF	ENCSR279MCN	bam	alignments	H3K36me3-human	1	1_1
esophagus	ENCFF192PWM	ENCSR034ZHF	bam	alignments	H3K36me3-human	1	1_1
H1	ENCFF805WZT	ENCSR496DCY	bam	alignments	H3K36me3-human	2	2_2
H1	ENCFF697AQU	ENCSR496DCY	bam	alignments	H3K36me3-human	1	1_1
H1	ENCFF295LHK	ENCSR476KTK	bam	alignments	H3K36me3-human	1	1_1
H1	ENCFF603HTP	ENCSR476KTK	bam	alignments	H3K36me3-human	2	2_1
H1	ENCFF619JFN	ENCSR925LJZ	bam	alignments	H3K36me3-human	1	1_1
H1	ENCFF044YAN	ENCSR925LJZ	bam	alignments	H3K36me3-human	2	2_1
mesenchymal stem cell	ENCFF412PPW	ENCSR824UNY	bam	alignments	H3K36me3-human	1	1_1
mesenchymal stem cell	ENCFF651ARP	ENCSR824UNY	bam	alignments	H3K36me3-human	2	2_1
mesendoderm	ENCFF077SHE	ENCSR144RXL	bam	alignments	H3K36me3-human	1	1_1
mesendoderm	ENCFF682KFP	ENCSR144RXL	bam	alignments	H3K36me3-human	2	2_1
mesodermal cell	ENCFF592HWS	ENCSR100LWU	bam	alignments	H3K36me3-human	1	1_4
mesodermal cell	ENCFF335KOW	ENCSR100LWU	bam	alignments	H3K36me3-human	2	2_2
neuronal stem cell	ENCFF095MCZ	ENCSR256ESY	bam	alignments	H3K36me3-human	1	1_1
neuronal stem cell	ENCFF964FXG	ENCSR256ESY	bam	alignments	H3K36me3-human	2	2_1
neuronal stem cell	ENCFF726UFJ	ENCSR238WMO	bam	alignments	H3K36me3-human	2	2_1
neuronal stem cell	ENCFF226NJT	ENCSR238WMO	bam	alignments	H3K36me3-human	1	1_1
pancreas	ENCFF693EDE	ENCSR943JOF	bam	alignments	H3K36me3-human	1	1_1
pancreas	ENCFF075MGN	ENCSR393HBQ	bam	alignments	H3K36me3-human	1	1_1
psoas muscle	ENCFF664YVG	ENCSR277PDE	bam	alignments	H3K36me3-human	1	1_2
sigmoid colon	ENCFF296GNA	ENCSR445RFF	bam	alignments	H3K36me3-human	1	1_1
sigmoid colon	ENCFF501JBW	ENCSR751JOQ	bam	alignments	H3K36me3-human	1	1_1
small intestine	ENCFF633TEH	ENCSR073YZL	bam	alignments	H3K36me3-human	1	1_1
small intestine	ENCFF674FLQ	ENCSR958DEW	bam	alignments	H3K36me3-human	1	1_1
small intestine	ENCFF739XVS	ENCSR205NEW	bam	alignments	H3K36me3-human	1	1_4
spleen	ENCFF011YZH	ENCSR466DUB	bam	alignments	H3K36me3-human	1	1_1
spleen	ENCFF232GTM	ENCSR466DUB	bam	alignments	H3K36me3-human	1	1_1
spleen	ENCFF361ULW	ENCSR078BHK	bam	alignments	H3K36me3-human	1	1_1
spleen	ENCFF259KHT	ENCSR078BHK	bam	alignments	H3K36me3-human	1	1_1
stomach	ENCFF814EYF	ENCSR697YSL	bam	alignments	H3K36me3-human	1	1_2
stomach	ENCFF749IJO	ENCSR269GMC	bam	alignments	H3K36me3-human	1	1_1
stomach	ENCFF731WJY	ENCSR552MZH	bam	alignments	H3K36me3-human	1	1_1
trophoblast cell	ENCFF079SNV	ENCSR038OIN	bam	alignments	H3K36me3-human	1	1_1
trophoblast cell	ENCFF763USC	ENCSR038OIN	bam	alignments	H3K36me3-human	2	2_1
trophoblast cell	ENCFF294APS	ENCSR005YZH	bam	alignments	H3K36me3-human	1	1_1
aorta	ENCFF176CXM	ENCSR957BPJ	bam	alignments	H3K4me3-human	1	1_1
aorta	ENCFF817YZO	ENCSR960EVO	bam	alignments	H3K4me3-human	1	1_2
CD4-positive, alpha-beta T cell	ENCFF681JNH	ENCSR263WLD	bam	alignments	H3K4me3-human	1	1_4
CD4-positive, alpha-beta T cell	ENCFF962FVV	ENCSR852FRR	bam	alignments	H3K4me3-human	1	1_2
CD8-positive, alpha-beta T cell	ENCFF470ZIH	ENCSR796CSH	bam	alignments	H3K4me3-human	1	1_1
endodermal cell	ENCFF347EDU	ENCSR446ZCY	bam	alignments	H3K4me3-human	1	1_5

Biosample term name	File accession	Experiment accession	File format	Output type	Experiment target	Biological replicate(s)	Technical replicate(s)
endodermal cell	ENCFF937ZPT	ENCSR446ZCY	bam	alignments	H3K4me3-human	2	2_5
esophagus	ENCFF894DUH	ENCSR697GPO	bam	alignments	H3K4me3-human	1	1_2
esophagus	ENCFF585DLK	ENCSR577ILY	bam	alignments	H3K4me3-human	1	1_1
H1	ENCFF467XCU	ENCSR019SQX	bam	alignments	H3K4me3-human	1	1_1
H1	ENCFF494FNC	ENCSR019SQX	bam	alignments	H3K4me3-human	2	2_1
H1	ENCFF640RPS	ENCSR019SQX	bam	alignments	H3K4me3-human	3	3_1
mesenchymal stem cell	ENCFF071ETA	ENCSR501JET	bam	alignments	H3K4me3-human	1	1_1
mesenchymal stem cell	ENCFF707CSG	ENCSR501JET	bam	alignments	H3K4me3-human	2	2_1
mesendoderm	ENCFF948LKM	ENCSR441SAT	bam	alignments	H3K4me3-human	1	1_2
mesendoderm	ENCFF072FDU	ENCSR441SAT	bam	alignments	H3K4me3-human	2	2_2
neuronal stem cell	ENCFF396QPN	ENCSR354XWM	bam	alignments	H3K4me3-human	1	1_2
neuronal stem cell	ENCFF490QCW	ENCSR354XWM	bam	alignments	H3K4me3-human	2	2_1
neuronal stem cell	ENCFF943BCG	ENCSR956CTX	bam	alignments	H3K4me3-human	1	1_1
neuronal stem cell	ENCFF102CQQ	ENCSR956CTX	bam	alignments	H3K4me3-human	2	2_1
pancreas	ENCFF056MDM	ENCSR747VED	bam	alignments	H3K4me3-human	1	1_1
pancreas	ENCFF907SLS	ENCSR315LPR	bam	alignments	H3K4me3-human	1	1_3
psoas muscle	ENCFF215MSA	ENCSR245BEV	bam	alignments	H3K4me3-human	1	1_1
psoas muscle	ENCFF959CCK	ENCSR949OYZ	bam	alignments	H3K4me3-human	1	1_2
sigmoid colon	ENCFF219BET	ENCSR321SZE	bam	alignments	H3K4me3-human	1	1_1
sigmoid colon	ENCFF917ZBE	ENCSR421HUB	bam	alignments	H3K4me3-human	1	1_2
small intestine	ENCFF883KQO	ENCSR237QFJ	bam	alignments	H3K4me3-human	1	1_2
small intestine	ENCFF035OFJ	ENCSR944QSH	bam	alignments	H3K4me3-human	1	1_1
small intestine	ENCFF070DOR	ENCSR792IJA	bam	alignments	H3K4me3-human	1	1_1
spleen	ENCFF346MPS	ENCSR432KIH	bam	alignments	H3K4me3-human	1	1_1
spleen	ENCFF266SHE	ENCSR432KIH	bam	alignments	H3K4me3-human	1	1_1
spleen	ENCFF205USG	ENCSR448FZC	bam	alignments	H3K4me3-human	1	1_1
spleen	ENCFF566BTS	ENCSR448FZC	bam	alignments	H3K4me3-human	1	1_1
stomach	ENCFF630XTQ	ENCSR202RXT	bam	alignments	H3K4me3-human	1	1_2
stomach	ENCFF937AGY	ENCSR129NCV	bam	alignments	H3K4me3-human	1	1_1
trophoblast cell	ENCFF638UNA	ENCSR874WOB	bam	alignments	H3K4me3-human	1	1_1
trophoblast cell	ENCFF961MFR	ENCSR874WOB	bam	alignments	H3K4me3-human	2	2_1
aorta	ENCFF371ZWQ	ENCSR065ZNA	bam	alignments	H3K9me3-human	1	1_1
CD4-positive, alpha-beta T cell	ENCFF616YFF	ENCSR453GNY	bam	alignments	H3K9me3-human	1	1_4
CD4-positive, alpha-beta T cell	ENCFF877MMM	ENCSR787WLV	bam	alignments	H3K9me3-human	1	1_2
CD8-positive, alpha-beta T cell	ENCFF953NDJ	ENCSR824PXG	bam	alignments	H3K9me3-human	1	1_3
ectodermal cell	ENCFF405EWU	ENCSR235CEI	bam	alignments	H3K9me3-human	1	1_2
endodermal cell	ENCFF520XOV	ENCSR823BHO	bam	alignments	H3K9me3-human	2	2_5
endodermal cell	ENCFF184CLR	ENCSR823BHO	bam	alignments	H3K9me3-human	1	1_5
esophagus	ENCFF189GCC	ENCSR150GLE	bam	alignments	H3K9me3-human	1	1_1
esophagus	ENCFF072NBC	ENCSR200WDD	bam	alignments	H3K9me3-human	1	1_1
H1	ENCFF421AGL	ENCSR395USV	bam	alignments	H3K9me3-human	2	2_2
H1	ENCFF008ALX	ENCSR395USV	bam	alignments	H3K9me3-human	1	1_1
H1	ENCFF597CRW	ENCSR883AQJ	bam	alignments	H3K9me3-human	1	1_2
H1	ENCFF354TVY	ENCSR883AQJ	bam	alignments	H3K9me3-human	2	2_1
H1	ENCFF913CWL	ENCSR883AQJ	bam	alignments	H3K9me3-human	3	3_1, 3_2
mesenchymal stem cell	ENCFF009ADE	ENCSR746CUY	bam	alignments	H3K9me3-human	1	1_1

Biosample term name	File accession	Experiment accession	File format	Output type	Experiment target	Biological replicate(s)	Technical replicate(s)
mesenchymal stem cell	ENCFF533ZII	ENCSR746CUY	bam	alignments	H3K9me3-human	2	2_1
mesodermal cell	ENCFF619CYH	ENCSR887ZPC	bam	alignments	H3K9me3-human	2	2_2
mesodermal cell	ENCFF334UGS	ENCSR887ZPC	bam	alignments	H3K9me3-human	3	3_2
neuronal stem cell	ENCFF483TNX	ENCSR391WDE	bam	alignments	H3K9me3-human	1	1_1
neuronal stem cell	ENCFF200TLD	ENCSR391WDE	bam	alignments	H3K9me3-human	2	2_1
neuronal stem cell	ENCFF369SMM	ENCSR800IIW	bam	alignments	H3K9me3-human	1	1_1
neuronal stem cell	ENCFF347GTB	ENCSR800IIW	bam	alignments	H3K9me3-human	2	2_1
pancreas	ENCFF705BUN	ENCSR533HDU	bam	alignments	H3K9me3-human	1	1_1
pancreas	ENCFF506OJR	ENCSR035QNZ	bam	alignments	H3K9me3-human	1	1_1
psoas muscle	ENCFF062DIE	ENCSR394DRL	bam	alignments	H3K9me3-human	1	1_1
sigmoid colon	ENCFF900CUX	ENCSR737NLJ	bam	alignments	H3K9me3-human	1	1_1
sigmoid colon	ENCFF046FUT	ENCSR636IDR	bam	alignments	H3K9me3-human	1	1_1
small intestine	ENCFF959CNM	ENCSR417RFS	bam	alignments	H3K9me3-human	1	1_1
small intestine	ENCFF036JLB	ENCSR773TWR	bam	alignments	H3K9me3-human	1	1_2
small intestine	ENCFF181EGL	ENCSR270VNK	bam	alignments	H3K9me3-human	1	1_1
spleen	ENCFF928TDB	ENCSR249XEB	bam	alignments	H3K9me3-human	1	1_1
spleen	ENCFF858WJB	ENCSR249XEB	bam	alignments	H3K9me3-human	1	1_1
spleen	ENCFF177AJJ	ENCSR421FPV	bam	alignments	H3K9me3-human	1	1_1
spleen	ENCFF694MHC	ENCSR421FPV	bam	alignments	H3K9me3-human	1	1_1
stomach	ENCFF606BDG	ENCSR885CMN	bam	alignments	H3K9me3-human	1	1_1
stomach	ENCFF642FEE	ENCSR639RKZ	bam	alignments	H3K9me3-human	1	1_1
stomach	ENCFF081KPW	ENCSR447GWQ	bam	alignments	H3K9me3-human	1	1_3
trophoblast cell	ENCFF220LTL	ENCSR044WCY	bam	alignments	H3K9me3-human	1	1_1
trophoblast cell	ENCFF932LWO	ENCSR044WCY	bam	alignments	H3K9me3-human	2	2_1
trophoblast cell	ENCFF915XKH	ENCSR044WCY	bam	alignments	H3K9me3-human	2	2_1

Supplementary Table S3: Metadata for the retrieved Human Epigenome Atlas ChIP-seq alignment files. For each biosample, the associated information include the file accession number, experiment accession number, file format and replicates are listed 5 ChIP-seq targets, including H3K27ac, H3K27me3, H3K36me3, H3K4me3 and H3K9me3.

Biosample term name	File accession	Experiment accession	File format	Output type	Experiment target	Biological replicate(s)	Technical replicate(s)
adipose tissue	ENCFF154CXY	ENCSR082SHT	bed narrowPeak	pseudo-replicated peaks	H3K27ac-human	1	1_1
aorta	ENCFF686UBD	ENCSR322TJD	bed narrowPeak	pseudo-replicated peaks	H3K27ac-human	1	1_2
aorta	ENCFF057LJS	ENCSR519CFV	bed narrowPeak	pseudo-replicated peaks	H3K27ac-human	2	2_1
CD4-positive, alpha-beta T cell	ENCFF901AMN	ENCSR546SDM	bed narrowPeak	pseudo-replicated peaks	H3K27ac-human	2	2_1
CD4-positive, alpha-beta T cell	ENCFF449ITG	ENCSR561KOM	bed narrowPeak	pseudo-replicated peaks	H3K27ac-human	1	1_2
CD8-positive, alpha-beta T cell	ENCFF321NYQ	ENCSR007HLH	bed narrowPeak	pseudo-replicated peaks	H3K27ac-human	1	1_2
CD8-positive, alpha-beta T cell	ENCFF243EDS	ENCSR835OJV	bed narrowPeak	replicated peaks	H3K27ac-human	1, 2	1_1, 2_1
ectodermal cell	ENCFF909CTU	ENCSR747HAM	bed narrowPeak	replicated peaks	H3K27ac-human	1, 2	1_2, 2_2
endodermal cell	ENCFF435HDB	ENCSR200ETW	bed narrowPeak	replicated peaks	H3K27ac-human	1, 2	1_2, 2_2
esophagus	ENCFF352GQN	ENCSR645SYH	bed narrowPeak	pseudo-replicated peaks	H3K27ac-human	1	1_1
esophagus	ENCFF535PRN	ENCSR679OVD	bed narrowPeak	pseudo-replicated peaks	H3K27ac-human	1	1_1
H1	ENCFF045CUG	ENCSR880SUY	bed narrowPeak	replicated peaks	H3K27ac-human	1, 2	1_2, 2_1
mesenchymal stem cell	ENCFF268FCW	ENCSR013KEC	bed narrowPeak	replicated peaks	H3K27ac-human	1, 2	1_1, 2_1
mesendoderm	ENCFF459UTL	ENCSR473PNT	bed narrowPeak	replicated peaks	H3K27ac-human	1, 2	1_1, 2_1
mesodermal cell	ENCFF997UPL	ENCSR931WLE	bed narrowPeak	replicated peaks	H3K27ac-human	1, 2	1_2, 2_2
neuronal stem cell	ENCFF722OBP	ENCSR799SRL	bed narrowPeak	replicated peaks	H3K27ac-human	1, 3	1_1, 3_1
pancreas	ENCFF064KHS	ENCSR402HFW	bed narrowPeak	pseudo-replicated peaks	H3K27ac-human	1	1_1
pancreas	ENCFF769XNX	ENCSR612BWE	bed narrowPeak	pseudo-replicated peaks	H3K27ac-human	1	1_1
psoas muscle	ENCFF779CYV	ENCSR250NHD	bed narrowPeak	pseudo-replicated peaks	H3K27ac-human	1	1_1
psoas muscle	ENCFF110BLF	ENCSR367WYJ	bed narrowPeak	pseudo-replicated peaks	H3K27ac-human	1	1_1, 1_2
psoas muscle	ENCFF265IZO	ENCSR791ISZ	bed narrowPeak	pseudo-replicated peaks	H3K27ac-human	2	2_1
sigmoid colon	ENCFF077GUP	ENCSR213SMK	bed narrowPeak	pseudo-replicated peaks	H3K27ac-human	1	1_1
sigmoid colon	ENCFF096HVY	ENCSR561YSH	bed narrowPeak	pseudo-replicated peaks	H3K27ac-human	1	1_1
small intestine	ENCFF581EXO	ENCSR454VRA	bed narrowPeak	pseudo-replicated peaks	H3K27ac-human	1	1_1
small intestine	ENCFF434KGU	ENCSR543CPW	bed narrowPeak	pseudo-replicated peaks	H3K27ac-human	1	1_3
small intestine	ENCFF484AHL	ENCSR655XLM	bed narrowPeak	pseudo-replicated peaks	H3K27ac-human	1	1_1
small intestine	ENCFF458TBT	ENCSR892XFG	bed narrowPeak	pseudo-replicated peaks	H3K27ac-human	1	1_2
spleen	ENCFF233IPB	ENCSR086XCT	bed narrowPeak	pseudo-replicated peaks	H3K27ac-human	1	1_1
spleen	ENCFF015DHA	ENCSR086XCT	bed narrowPeak	pseudo-replicated peaks	H3K27ac-human	1	1_1
spleen	ENCFF158FZE	ENCSR170MAJ	bed narrowPeak	pseudo-replicated peaks	H3K27ac-human	1	1_1
spleen	ENCFF273NFQ	ENCSR170MAJ	bed narrowPeak	pseudo-replicated peaks	H3K27ac-human	1	1_1
spleen	ENCFF661WLX	ENCSR235ZBF	bed narrowPeak	pseudo-replicated peaks	H3K27ac-human	1	1_2
spleen	ENCFF145NKN	ENCSR235ZBF	bed narrowPeak	pseudo-replicated peaks	H3K27ac-human	1	1_2
stomach	ENCFF598JDA	ENCSR001SHB	bed narrowPeak	pseudo-replicated peaks	H3K27ac-human	1	1_2
stomach	ENCFF055FKA	ENCSR437QMD	bed narrowPeak	pseudo-replicated peaks	H3K27ac-human	1	1_1, 1_2
stomach	ENCFF268OSV	ENCSR582UTE	bed narrowPeak	pseudo-replicated peaks	H3K27ac-human	1	1_1
stomach	ENCFF878SVN	ENCSR743DDX	bed narrowPeak	pseudo-replicated peaks	H3K27ac-human	1	1_2
trophoblast	ENCFF972AZZ	ENCSR507SRD	bed narrowPeak	pseudo-replicated peaks	H3K27ac-human	• 1	1_1
trophoblast	ENCFF832MMQ	ENCSR955XFL	bed narrowPeak	pseudo-replicated peaks	H3K27ac-human	1	1_1
aorta	ENCFF887VHI	ENCSR128VHV	bed narrowPeak	pseudo-replicated peaks	H3K27me3-human	1	1_2
aorta	ENCFF634IHR	ENCSR196PGM	bed narrowPeak	pseudo-replicated peaks	H3K27me3-human	1	1_1
CD4-positive, alpha-beta T cell	ENCFF614QJJ	ENCSR043SBG	bed narrowPeak	pseudo-replicated peaks	H3K27me3-human	1	1_4
CD4-positive, alpha-beta T cell	ENCFF310UPS	ENCSR043SBG	bed narrowPeak	replicated peaks	H3K27me3-human	1, 2	1_4, 2_2
CD4-positive, alpha-beta T cell	ENCFF211ERK	ENCSR733QOZ	bed narrowPeak	pseudo-replicated peaks	H3K27me3-human	1	1_2
CD8-positive, alpha-beta T cell	ENCFF565COF	ENCSR103GGR	bed narrowPeak	pseudo-replicated peaks	H3K27me3-human	1	1_4
CD8-positive, alpha-beta T cell	ENCFF200PGJ	ENCSR639HVJ	bed narrowPeak	pseudo-replicated peaks	H3K27me3-human	1	1_1

Biosample term name	File accession	Experiment accession	File format	Output type	Experiment target	Biological replicate(s)	Technical replicate(s)
CD8-positive, alpha-beta T cell	ENCFF515XPE	ENCSR797GOJ	bed narrowPeak	replicated peaks	H3K27me3-human	1, 2	1_1, 2_1
ectodermal cell	ENCFF940CGE	ENCSR690GLT	bed narrowPeak	replicated peaks	H3K27me3-human	1, 3	1_2, 3_2
endodermal cell	ENCFF901CSA	ENCSR273IYV	bed narrowPeak	replicated peaks	H3K27me3-human	1, 2	1_5, 2_5
esophagus	ENCFF472RYD	ENCSR088GXB	bed narrowPeak	pseudo-replicated peaks	H3K27me3-human	1	1_1
esophagus	ENCFF033JBF	ENCSR641RQV	bed narrowPeak	pseudo-replicated peaks	H3K27me3-human	1	1_2
H1	ENCFF156RHD	ENCSR186OBR	bed narrowPeak	replicated peaks	H3K27me3-human	1, 2	1_1, 2_1
H1	ENCFF084QDP	ENCSR216OGD	bed narrowPeak	replicated peaks	H3K27me3-human	1, 2	1_1, 2_1
H1	ENCFF098JFF	ENCSR687FDK	bed narrowPeak	pseudo-replicated peaks	H3K27me3-human	1	1_1
H1	ENCFF411ESN	ENCSR928HYM	bed narrowPeak	replicated peaks	H3K27me3-human	1, 2	1_2, 2_1
mesenchymal stem cell	ENCFF541LUL	ENCSR262VXI	bed narrowPeak	replicated peaks	H3K27me3-human	1, 2	1_1, 2_1
mesenchymal stem cell	ENCFF759ILD	ENCSR832JVP	bed narrowPeak	replicated peaks	H3K27me3-human	1, 2	1_1, 2_1
mesendoderm	ENCFF273OXZ	ENCSR405AXO	bed narrowPeak	replicated peaks	H3K27me3-human	1, 2	1_1, 2_1
neuronal stem cell	ENCFF491GSQ	ENCSR550XZG	bed narrowPeak	replicated peaks	H3K27me3-human	1, 2	1_1, 2_1
neuronal stem cell	ENCFF504BOD	ENCSR692CTK	bed narrowPeak	replicated peaks	H3K27me3-human	1, 2	1_1, 2_1
neuronal stem cell	ENCFF044KMT	ENCSR694LBI	bed narrowPeak	replicated peaks	H3K27me3-human	1, 2	1_1, 2_1
pancreas	ENCFF581PLO	ENCSR186QKH	bed narrowPeak	pseudo-replicated peaks	H3K27me3-human	1	1_1
pancreas	ENCFF398GCM	ENCSR486NDF	bed narrowPeak	pseudo-replicated peaks	H3K27me3-human	1	1_1, 1_2
psoas muscle	ENCFF593RXH	ENCSR720SAS	bed narrowPeak	pseudo-replicated peaks	H3K27me3-human	1	1_1
psoas muscle	ENCFF285KGM	ENCSR843KHS	bed narrowPeak	pseudo-replicated peaks	H3K27me3-human	1	1_2
sigmoid colon	ENCFF199HGP	ENCSR042RIW	bed narrowPeak	pseudo-replicated peaks	H3K27me3-human	1	1_1
sigmoid colon	ENCFF145YBF	ENCSR897TGR	bed narrowPeak	pseudo-replicated peaks	H3K27me3-human	1	1_1
small intestine	ENCFF128PPS	ENCSR340OPI	bed narrowPeak	pseudo-replicated peaks	H3K27me3-human	1	1_1
small intestine	ENCFF321PQU	ENCSR859EIX	bed narrowPeak	pseudo-replicated peaks	H3K27me3-human	1	1_2
small intestine	ENCFF954AUE	ENCSR877PAS	bed narrowPeak	pseudo-replicated peaks	H3K27me3-human	1	1_2
spleen	ENCFF948PIU	ENCSR408ONP	bed narrowPeak	pseudo-replicated peaks	H3K27me3-human	1	1_1
spleen	ENCFF492VAT	ENCSR408ONP	bed narrowPeak	pseudo-replicated peaks	H3K27me3-human	1	1_1
spleen	ENCFF218HXK	ENCSR608FDQ	bed narrowPeak	pseudo-replicated peaks	H3K27me3-human	1	1_2
spleen	ENCFF065SNS	ENCSR608FDQ	bed narrowPeak	pseudo-replicated peaks	H3K27me3-human	1	1_2
stomach	ENCFF787QYF	ENCSR354IST	bed narrowPeak	pseudo-replicated peaks	H3K27me3-human	1	1_2
stomach	ENCFF464IGY	ENCSR527BFF	bed narrowPeak	pseudo-replicated peaks	H3K27me3-human	1	1_1
trophoblast	ENCFF027NPI	ENCSR221ZRM	bed narrowPeak	pseudo-replicated peaks	H3K27me3-human	1	1_1
trophoblast	ENCFF740AIH	ENCSR495OEG	bed narrowPeak	pseudo-replicated peaks	H3K27me3-human	1	1_1
aorta	ENCFF220HID	ENCSR673JYT	bed narrowPeak	pseudo-replicated peaks	H3K36me3-human	1	1_1
aorta	ENCFF695OJS	ENCSR989AMI	bed narrowPeak	pseudo-replicated peaks	H3K36me3-human	1	1_1
CD4-positive, alpha-beta T cell	ENCFF062JZY	ENCSR774OKQ	bed narrowPeak	pseudo-replicated peaks	H3K36me3-human	1	1_3
CD4-positive, alpha-beta T cell	ENCFF670MKJ	ENCSR828WZG	bed narrowPeak	pseudo-replicated peaks	H3K36me3-human	1	1_4
CD4-positive, alpha-beta T cell	ENCFF687BTK	ENCSR828WZG	bed narrowPeak	replicated peaks	H3K36me3-human	1, 2	1_4, 2_2
CD8-positive, alpha-beta T cell	ENCFF190SHZ	ENCSR681OSD	bed narrowPeak	pseudo-replicated peaks	H3K36me3-human	1	1_1
CD8-positive, alpha-beta T cell	ENCFF430GVA	ENCSR694CDP	bed narrowPeak	replicated peaks	H3K36me3-human	1, 2	1_1, 2_1
CD8-positive, alpha-beta T cell	ENCFF865NBY	ENCSR782NOO	bed narrowPeak	pseudo-replicated peaks	H3K36me3-human	1	1_3
endodermal cell	ENCFF280ASK	ENCSR677EZB	bed narrowPeak	replicated peaks	H3K36me3-human	1, 3	1_4, 3_2
esophagus	ENCFF693ZIC	ENCSR034ZHF	bed narrowPeak	pseudo-replicated peaks	H3K36me3-human	1	1_1
esophagus	ENCFF878XQI	ENCSR279MCN	bed narrowPeak	pseudo-replicated peaks	H3K36me3-human	1	1_1
H1	ENCFF093YFN	ENCSR476KTK	bed narrowPeak	replicated peaks	H3K36me3-human	1, 2	1_1, 2_1
H1	ENCFF422MTS	ENCSR496DCY	bed narrowPeak	replicated peaks	H3K36me3-human	1, 2	1_1, 2_2
H1	ENCFF736WAN	ENCSR925LJZ	bed narrowPeak	replicated peaks	H3K36me3-human	1, 2	1_1, 2_1

Biosample term name	File accession	Experiment accession	File format	Output type	Experiment target	Biological replicate(s)	Technical replicate(s)
mesenchymal stem cell	ENCFF115LHE	ENCSR555QHZ	bed narrowPeak	replicated peaks	H3K36me3-human	2, 3	2_1, 3_1
mesenchymal stem cell	ENCFF051RNB	ENCSR824UNY	bed narrowPeak	replicated peaks	H3K36me3-human	1, 2	1_1, 2_1
mesendoderm	ENCFF771IYF	ENCSR144RXL	bed narrowPeak	replicated peaks	H3K36me3-human	1, 2	1_1, 2_1
mesodermal cell	ENCFF049SYA	ENCSR100LWU	bed narrowPeak	replicated peaks	H3K36me3-human	1, 2	1_4, 2_2
neuronal stem cell	ENCFF286UMF	ENCSR238WMO	bed narrowPeak	replicated peaks	H3K36me3-human	1, 2	1_1, 2_1
neuronal stem cell	ENCFF999NTP	ENCSR256ESY	bed narrowPeak	replicated peaks	H3K36me3-human	1, 2	1_1, 2_1
pancreas	ENCFF752OPF	ENCSR393HBQ	bed narrowPeak	pseudo-replicated peaks	H3K36me3-human	1	1_1
pancreas	ENCFF987DRQ	ENCSR943JOF	bed narrowPeak	pseudo-replicated peaks	H3K36me3-human	1	1_1
psoas muscle	ENCFF209YRO	ENCSR277PDE	bed narrowPeak	pseudo-replicated peaks	H3K36me3-human	1	1_2
sigmoid colon	ENCFF567DCF	ENCSR445RFF	bed narrowPeak	pseudo-replicated peaks	H3K36me3-human	1	1_1
sigmoid colon	ENCFF332VLM	ENCSR751JOQ	bed narrowPeak	pseudo-replicated peaks	H3K36me3-human	1	1_1
small intestine	ENCFF599NYJ	ENCSR073YZL	bed narrowPeak	pseudo-replicated peaks	H3K36me3-human	1	1_1
small intestine	ENCFF842DWY	ENCSR205NEW	bed narrowPeak	pseudo-replicated peaks	H3K36me3-human	1	1_4
small intestine	ENCFF384JUV	ENCSR958DEW	bed narrowPeak	pseudo-replicated peaks	H3K36me3-human	1	1_1
spleen	ENCFF294HWV	ENCSR078BHK	bed narrowPeak	pseudo-replicated peaks	H3K36me3-human	1	1_1
spleen	ENCFF206EGT	ENCSR078BHK	bed narrowPeak	pseudo-replicated peaks	H3K36me3-human	1	1_1
spleen	ENCFF224MXB	ENCSR466DUB	bed narrowPeak	pseudo-replicated peaks	H3K36me3-human	1	1_1
spleen	ENCFF462KMA	ENCSR466DUB	bed narrowPeak	pseudo-replicated peaks	H3K36me3-human	1	1_1
stomach	ENCFF435RTF	ENCSR269GMC	bed narrowPeak	pseudo-replicated peaks	H3K36me3-human	1	1_1
stomach	ENCFF845PNV	ENCSR697YSL	bed narrowPeak	pseudo-replicated peaks	H3K36me3-human	1	1_2
trophoblast	ENCFF584XPR	ENCSR482KJD	bed narrowPeak	pseudo-replicated peaks	H3K36me3-human	1	1_1
trophoblast	ENCFF493PZU	ENCSR746MTU	bed narrowPeak	pseudo-replicated peaks	H3K36me3-human	1	1_1
aorta	ENCFF223WWD	ENCSR957BPJ	bed narrowPeak	pseudo-replicated peaks	H3K4me3-human	1	1_1
aorta	ENCFF455KIC	ENCSR960EVO	bed narrowPeak	pseudo-replicated peaks	H3K4me3-human	1	1_2
CD4-positive, alpha-beta T cell	ENCFF736VPN	ENCSR263WLD	bed narrowPeak	pseudo-replicated peaks	H3K4me3-human	1	1_4
CD4-positive, alpha-beta T cell	ENCFF093RDC	ENCSR263WLD	bed narrowPeak	replicated peaks	H3K4me3-human	1, 2	1_4, 2_2
CD4-positive, alpha-beta T cell	ENCFF674ZVF	ENCSR852FRR	bed narrowPeak	pseudo-replicated peaks	H3K4me3-human	1	1_2
CD8-positive, alpha-beta T cell	ENCFF269CAD	ENCSR166ZZZ	bed narrowPeak	pseudo-replicated peaks	H3K4me3-human	1	1_1
CD8-positive, alpha-beta T cell	ENCFF820IRX	ENCSR231FDF	bed narrowPeak	replicated peaks	H3K4me3-human	1, 2	1_1, 2_1
CD8-positive, alpha-beta T cell	ENCFF295CWC	ENCSR660KHZ	bed narrowPeak	pseudo-replicated peaks	H3K4me3-human	1	1_1
CD8-positive, alpha-beta T cell	ENCFF958QRD	ENCSR796CSH	bed narrowPeak	pseudo-replicated peaks	H3K4me3-human	1	1_1
ectodermal cell	ENCFF450NCT	ENCSR807LJO	bed narrowPeak	replicated peaks	H3K4me3-human	1, 3	1_2, 3_2
endodermal cell	ENCFF599VIR	ENCSR446ZCY	bed narrowPeak	replicated peaks	H3K4me3-human	1, 2	1_5, 2_5
esophagus	ENCFF812UXQ	ENCSR577ILY	bed narrowPeak	pseudo-replicated peaks	H3K4me3-human	1	1_1
esophagus	ENCFF232JVH	ENCSR697GPO	bed narrowPeak	pseudo-replicated peaks	H3K4me3-human	1	1_2
H1	ENCFF744ORJ	ENCSR003SSR	bed narrowPeak	replicated peaks	H3K4me3-human	1, 2	1_1, 2_1
H1	ENCFF408FCY	ENCSR019SQX	bed narrowPeak	replicated peaks	H3K4me3-human	1, 2	1_1, 2_1
H1	ENCFF277AOQ	ENCSR443YAS	bed narrowPeak	replicated peaks	H3K4me3-human	1, 2	1_1, 2_1
mesenchymal stem cell	ENCFF493ZYQ	ENCSR004AKD	bed narrowPeak	replicated peaks	H3K4me3-human	1, 2	1_1, 2_1
mesenchymal stem cell	ENCFF011GWS	ENCSR501JET	bed narrowPeak	replicated peaks	H3K4me3-human	1, 2	1_1, 2_1
mesendoderm	ENCFF052VWO	ENCSR441SAT	bed narrowPeak	replicated peaks	H3K4me3-human	1, 2	1_2, 2_2
mesodermal cell	ENCFF870OTW	ENCSR959RHF	bed narrowPeak	replicated peaks	H3K4me3-human	1, 2	1_2, 2_2
neuronal stem cell	ENCFF043FGL	ENCSR354XWM	bed narrowPeak	replicated peaks	H3K4me3-human	1, 2	1_2, 2_1
neuronal stem cell	ENCFF480IBO	ENCSR956CTX	bed narrowPeak	replicated peaks	H3K4me3-human	1, 2	1_1, 2_1
pancreas	ENCFF918CGC	ENCSR315LPR	bed narrowPeak	pseudo-replicated peaks	H3K4me3-human	1	1_3
pancreas	ENCFF147VOD	ENCSR747VED	bed narrowPeak	pseudo-replicated peaks	H3K4me3-human	1	1_1

Biosample term name	File accession	Experiment accession	File format	Output type	Experiment target	Biological replicate(s)	Technical replicate(s)
psoas muscle	ENCFF925HIR	ENCSR245BEV	bed narrowPeak	pseudo-replicated peaks	H3K4me3-human	1	1_1
psoas muscle	ENCFF783HFC	ENCSR949OYZ	bed narrowPeak	pseudo-replicated peaks	H3K4me3-human	1	1_2
sigmoid colon	ENCFF890JZD	ENCSR321SZE	bed narrowPeak	pseudo-replicated peaks	H3K4me3-human	1	1_1
sigmoid colon	ENCFF197CZF	ENCSR421HUB	bed narrowPeak	pseudo-replicated peaks	H3K4me3-human	1	1_2
small intestine	ENCFF012TFR	ENCSR237QFJ	bed narrowPeak	pseudo-replicated peaks	H3K4me3-human	1	1_2
small intestine	ENCFF563IRG	ENCSR792IJA	bed narrowPeak	pseudo-replicated peaks	H3K4me3-human	1	1_1
small intestine	ENCFF213LSU	ENCSR944QSH	bed narrowPeak	pseudo-replicated peaks	H3K4me3-human	1	1_1
spleen	ENCFF590BSY	ENCSR432KIH	bed narrowPeak	pseudo-replicated peaks	H3K4me3-human	1	1_1
spleen	ENCFF814FPR	ENCSR432KIH	bed narrowPeak	pseudo-replicated peaks	H3K4me3-human	1	1_1
spleen	ENCFF772AFU	ENCSR448FZC	bed narrowPeak	pseudo-replicated peaks	H3K4me3-human	1	1_1
spleen	ENCFF441DWZ	ENCSR448FZC	bed narrowPeak	pseudo-replicated peaks	H3K4me3-human	1	1_1
stomach	ENCFF805ZSK	ENCSR129NCV	bed narrowPeak	pseudo-replicated peaks	H3K4me3-human	1	1_1
stomach	ENCFF291FKK	ENCSR202RXT	bed narrowPeak	pseudo-replicated peaks	H3K4me3-human	1	1_2
aorta	ENCFF978QLH	ENCSR065ZNA	bed narrowPeak	pseudo-replicated peaks	H3K9me3-human	1	1_1
CD4-positive, alpha-beta T cell	ENCFF326XIB	ENCSR453GNY	bed narrowPeak	pseudo-replicated peaks	H3K9me3-human	1	1_4
CD4-positive, alpha-beta T cell	ENCFF255BCT	ENCSR453GNY	bed narrowPeak	replicated peaks	H3K9me3-human	1, 2	1_4, 2_2
CD4-positive, alpha-beta T cell	ENCFF323MFM	ENCSR787WLV	bed narrowPeak	pseudo-replicated peaks	H3K9me3-human	1	1_2
CD8-positive, alpha-beta T cell	ENCFF668CHY	ENCSR815PSO	bed narrowPeak	pseudo-replicated peaks	H3K9me3-human	1	1_1
CD8-positive, alpha-beta T cell	ENCFF552VSA	ENCSR824PXG	bed narrowPeak	pseudo-replicated peaks	H3K9me3-human	1	1_3
CD8-positive, alpha-beta T cell	ENCFF320HLH	ENCSR905SHH	bed narrowPeak	replicated peaks	H3K9me3-human	1, 2	1_1, 2_1
ectodermal cell	ENCFF482VRI	ENCSR235CEI	bed narrowPeak	replicated peaks	H3K9me3-human	1, 3	1_2, 3_2
endodermal cell	ENCFF446HIG	ENCSR823BHO	bed narrowPeak	replicated peaks	H3K9me3-human	1, 2	1_5, 2_5
esophagus	ENCFF040EBA	ENCSR150GLE	bed narrowPeak	pseudo-replicated peaks	H3K9me3-human	1	1_1
esophagus	ENCFF277ETB	ENCSR200WDD	bed narrowPeak	pseudo-replicated peaks	H3K9me3-human	1	1_1
H1	ENCFF250GSY	ENCSR395USV	bed narrowPeak	replicated peaks	H3K9me3-human	1, 2	1_1, 2_2
H1	ENCFF348GGB	ENCSR883AQJ	bed narrowPeak	replicated peaks	H3K9me3-human	1, 3	1_2, 3_1, 3_2
mesenchymal stem cell	ENCFF188ZUZ	ENCSR439EHQ	bed narrowPeak	replicated peaks	H3K9me3-human	1, 2	1_1, 2_1
mesenchymal stem cell	ENCFF022VRW	ENCSR746CUY	bed narrowPeak	replicated peaks	H3K9me3-human	1, 2	1_1, 2_1
mesodermal cell	ENCFF708SUO	ENCSR887ZPC	bed narrowPeak	replicated peaks	H3K9me3-human	2, 3	2_2, 3_2
neuronal stem cell	ENCFF718CGL	ENCSR391WDE	bed narrowPeak	replicated peaks	H3K9me3-human	1, 2	1_1, 2_1
neuronal stem cell	ENCFF304FHZ	ENCSR800IIW	bed narrowPeak	replicated peaks	H3K9me3-human	1, 2	1_1, 2_1
pancreas	ENCFF501UUF	ENCSR035QNZ	bed narrowPeak	pseudo-replicated peaks	H3K9me3-human	1	1_1
pancreas	ENCFF153MCW	ENCSR533HDU	bed narrowPeak	pseudo-replicated peaks	H3K9me3-human	1	1_1
psoas muscle	ENCFF368EHI	ENCSR394DRL	bed narrowPeak	pseudo-replicated peaks	H3K9me3-human	1	1_1
sigmoid colon	ENCFF824HBO	ENCSR636IDR	bed narrowPeak	pseudo-replicated peaks	H3K9me3-human	1	1_1
sigmoid colon	ENCFF417EMO	ENCSR737NLJ	bed narrowPeak	pseudo-replicated peaks	H3K9me3-human	1	1_1
small intestine	ENCFF482VIL	ENCSR270VNK	bed narrowPeak	pseudo-replicated peaks	H3K9me3-human	1	1_1
small intestine	ENCFF303WRT	ENCSR417RFS	bed narrowPeak	pseudo-replicated peaks	H3K9me3-human	1	1_1
small intestine	ENCFF274KHB	ENCSR773TWR	bed narrowPeak	pseudo-replicated peaks	H3K9me3-human	1	1_2
spleen	ENCFF561FFX	ENCSR249XEB	bed narrowPeak	pseudo-replicated peaks	H3K9me3-human	1	1_1
spleen	ENCFF688HWH	ENCSR249XEB	bed narrowPeak	pseudo-replicated peaks	H3K9me3-human	1	1_1
spleen	ENCFF932LXW	ENCSR421FPV	bed narrowPeak	pseudo-replicated peaks	H3K9me3-human	1	1_1
spleen	ENCFF424ZSF	ENCSR421FPV	bed narrowPeak	pseudo-replicated peaks	H3K9me3-human	1	1_1
stomach	ENCFF957ZGX	ENCSR447GWQ	bed narrowPeak	pseudo-replicated peaks	H3K9me3-human	1	1_3
stomach	ENCFF822CXT	ENCSR639RKZ	bed narrowPeak	pseudo-replicated peaks	H3K9me3-human	1	1_1
stomach	ENCFF496LZT	ENCSR885CMN	bed narrowPeak	pseudo-replicated peaks	H3K9me3-human	1	1_1

Biosample term name	File accession	Experiment accession	File format	Output type	Experiment target	Biological replicate(s)	Technical replicate(s)
trophoblast	ENCFF309GOP	ENCSR356JTB	bed narrowPeak	pseudo-replicated peaks	H3K9me3-human	1	1_1
trophoblast	ENCFF335ZGF	ENCSR612MZB	bed narrowPeak	pseudo-replicated peaks	H3K9me3-human	1	1_1

Supplementary Table S4: Metadata for the retrieved Human Epigenome Atlas ChIP-seq peak files. For each biosample, the associated information include the file accession number, experiment accession number, file format and replicates are listed 5 ChIP-seq targets, including H3K27ac, H3K27me3, H3K36me3, H3K4me3 and H3K9me3.

Symbol	Gene description
**A2ML1**	alpha-2-macroglobulin like 1
**COL11A2**	collagen type XI alpha 2 chain
**DNAH1**	dynein axonemal heavy chain 1
**DNAH10**	dynein axonemal heavy chain 10
**DNAH9**	dynein axonemal heavy chain 9
**HYDIN**	HYDIN axonemal central pair apparatus protein
**IPO5**	importin 5
**JAKMIP1**	janus kinase and microtubule interacting protein 1
**MYO1H**	myosin IH
**NEB**	nebulin
**RANBP17**	RAN binding protein 17
**TRPM5**	transient receptor potential cation channel subfamily M member 5
**USHBP1**	USH1 protein network component harmonin binding protein 1
**VWA5B1**	von Willebrand factor A domain containing 5B1

Supplementary Table S5: List of genes with odds ratio > 1 and Fisher Exact Test adjusted $p - value \leq 0.05$

	DEU	H3K27ac	H3K27me3	H3K36me3	H3K4me3	H3K9me3
**Potency**	0.091	0.481	0.423	0.056	0.052	0.105
**Type**	0.177	0.379	0.130	0.048	0.162	0.118
**Origin**	0.459	0.420	0.176	0.022	0.200	0.152
**Life stage**	0.016	0.304	0.238	0.031	0.033	0.029

Supplementary Table S6: Adjusted Rand indices measuring the similarity between differential features-based hierarchical clustering based on and tissue label schemes. Investigated cell types were separated by potency, sample type, origin and life stage and compared to the cluster labels from hierarchical clustering.
